# Genetically encoded protein photocrosslinker with a transferable mass spectrometry-identifiable label

**DOI:** 10.1038/ncomms12299

**Published:** 2016-07-27

**Authors:** Yi Yang, Haiping Song, Dan He, Shuai Zhang, Shizhong Dai, Shixian Lin, Rong Meng, Chu Wang, Peng R. Chen

**Affiliations:** 1 Synthetic and Functional Biomolecules Center, Beijing National Laboratory for Molecular Sciences, Key Laboratory of Bioorganic Chemistry and Molecular Engineering of Ministry of Education, Department of Chemical Biology, College of Chemistry and Molecular Engineering, Peking University, Beijing 100871, China; 2 Peking-Tsinghua Center for Life Sciences, Beijing 100871, China

## Abstract

Coupling photocrosslinking reagents with mass spectrometry has become a powerful tool for studying protein–protein interactions in living systems, but it still suffers from high rates of false-positive identifications as well as the lack of information on interaction interface due to the challenges in deciphering crosslinking peptides. Here we develop a genetically encoded photo-affinity unnatural amino acid that introduces a mass spectrometry-identifiable label (MS-label) to the captured prey proteins after photocrosslinking and prey–bait separation. This strategy, termed IMAPP (*In-situ* cleavage and MS-label transfer After Protein Photocrosslinking), enables direct identification of photo-captured substrate peptides that are difficult to uncover by conventional genetically encoded photocrosslinkers. Taking advantage of the MS-label, the IMAPP strategy significantly enhances the confidence for identifying protein–protein interactions and enables simultaneous mapping of the binding interface under living conditions.

## Abstract (web summary)

Mapping protein-protein interaction using crosslinking and mass spectroscopy strategies is hampered by a high rate of false-positive results. Here, the authors develop a genetically encoded photo-affinity probe for accurate identification of protein interaction partners and crosslinking sites.

## Graphical abstract

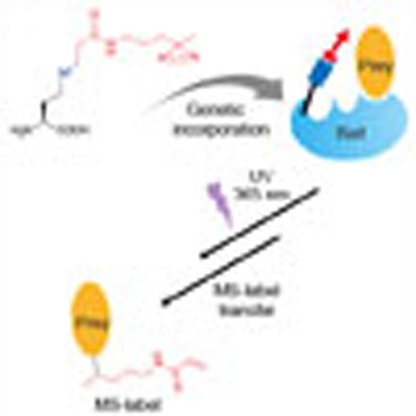

Techniques for discovering and characterizing protein–protein interactions under physiological conditions are under constant development, among which chemical and photo-affinity crosslinking strategies have drawn increasing attention in recent years^1^,^2^,^3^,^4^,^5^. The ability of converting non-covalent interactions between biomolecules into covalent linkages allows capture of weak and transient protein–protein interactions frequently found in nature^3^,^4^,^5^,^6^,^7^. Genetically encoded photocrosslinkers, typically in the form of photo-affinity ‘unnatural amino acids' (UAAs), can be incorporated into proteins at desired positions via the genetic code expansion strategy^8^. This method uses an orthogonal aminoacyl-transfer RNA (tRNA) synthetase (aaRS)-tRNA pair to incorporate the desired UAA such as a photocrosslinker in response to an in-frame amber codon in the target gene, allowing site-specific photocrosslinking for capturing protein–protein interactions under living conditions^4^,^5^,^8^,^9^,^10^,^11^,^12^,^13^,^14^. However, the downstream procedures for target separation and identification still follow regular affinity pull-down protocols that suffer from the problem of false identification due to non-specific protein binding and/or indirect protein interactions^10^,^15^,^16^,^17^. More importantly, the crosslinking peptides and modification sites are difficult to uncover by current photocrosslinkers, which would otherwise provide valuable information regarding the interaction interface^4^,^12^.

We envision that these limitations can be overcome by integrating a stable transferable mass spectrometry-identifiable label (MS-label) into the photo-affinity probe, which can be subsequently transferred to the crosslinked prey proteins through a cleavage linker after protein photocrosslinking^18^,^19^,^20^. By searching the MS-label modified peptides, the crosslinked interacting proteins can be readily distinguished from the background, thus improving the specificity, confidence as well as robustness of the target identification process^21^,^22^,^23^. Meanwhile, the MS-label modified peptides can provide structural information of interaction interface^4^. Herein, we report the design and development of such a genetically encoded photo-affinity UAA that contains a cleavable linker for prey–bait separation and an *in situ* generated MS-label that can be transferred to the prey proteins upon cleavage. Embarked on this unique photocrosslinker, we create a novel chemical proteomic strategy, termed ‘IMAPP' (*In-situ* cleavage and MS-label transfer After Protein Photocrosslinking), that enables simultaneous identification of the captured peptides and the exact crosslinking sites, which is highly valuable for uncovering target proteins as well as mapping protein–protein interaction interfaces under living conditions.

## Results

### Design and characterization of DiZHSeC

**Figure 1:**
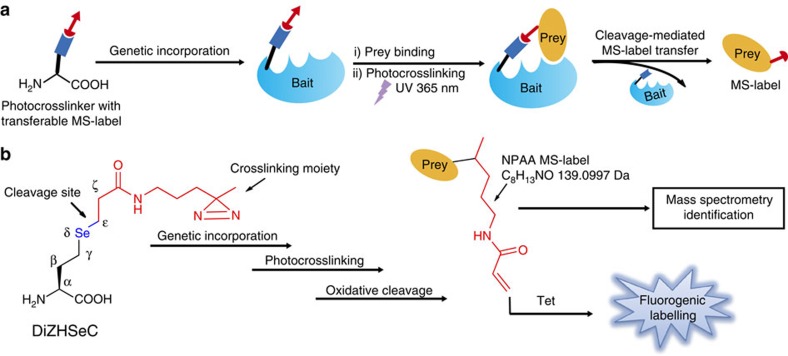
Development of genetically encoded photocrosslinker with a transferable MS-label. (**a**) *In situ* generation of MS-label on prey proteins by using a genetically encoded cleavable photocrosslinker. (**b**) Chemical design of the photocrosslinker (DiZHSeC) with transferable MS-label. The *in situ* generated NPAA MS-label can be verified by either fluorogenic labelling or can be directly identified by mass spectrometry.

We have recently developed a genetically encoded photocrosslinker-DiZPK containing the diazirine group that can be used for highly efficient photo-affinity capture of protein–protein interactions in living systems^14^. By replacing the γ-carbon with a selenium (Se) atom, DiZPK can be further converted to a cleavable photocrosslinker–DiZSeK in which the selenium–carbon bond can undergo oxidative cleavage upon H_2_O_2_ treatment, facilitating the prey–bait separation as well as the downstream target identification^19^. Both these UAAs are pyrrolysine (Pyl) analogues that can be specifically recognized by a mutant pyrrolysyl-tRNA synthetase (PylRS) with its cognitive 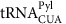 from archaea species such as *Methanosarcina barkeri* (*Mb*)^8^,^14^,^24^,^25^,^26^. However, the selenenic acid moiety generated after oxidative cleavage on the prey proteins is too labile for MS identification and, therefore, cannot serve as a stable MS-label. To overcome this limitation, we herein designed an alternative UAA structure by replacing the δ-carbon on DiZPK by a Se atom and changing the ζ-amine to a methylene group. The resulting photocrosslinker (Se-(N-(3-(3-methyl-3H-diazirin-3-yl)propyl)propanamide)-3-yl-homoselenocysteine), named as DiZHSeC, can undergo the oxidation-mediated C_ɛ_-Se_δ_ bond cleavage and produce an N-(4,4-bis-substituted-pentyl)acrylamide (NPAA) moiety that is stable and readily identifiable by mass spectrometry (Fig. 1a,b). We synthesized DiZHSeC according to the procedure shown in Supplementary Fig. 1 and monitored its oxidative cleavage by ultra-performance liquid chromatography mass spectrometry. Upon treatment with 8 mM H_2_O_2_ for 60 min, we observed full cleavage of the C_ɛ_-Se_δ_ bond in DiZHSeC and the generation of a new peak corresponding to the cleaved product P1 containing the NPAA moiety (Supplementary Fig. 2). As expected, the resulting acrylamide group from H_2_O_2_-mediated cleavage survived through the oxidative condition and remained intact, making it a stable tag suitable for chemical derivatization and MS identification.

### Site-specific encoding of DiZHSeC in *Escherichia coli*

**Figure 2:**
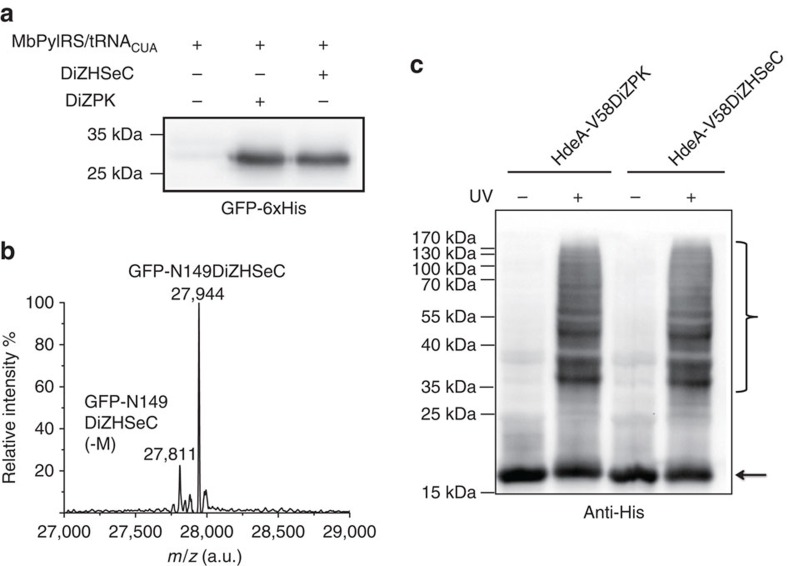
Site-specific incorporation of DiZHSeC into proteins in *E. coli*. (**a**) Immunoblotting analysis shows similar amber suppression efficiency of the DiZPK-recognizing PylRS mutant for DiZPK and DiZHSeC when they are inserted to N149 position of GFP (GFP-N149TAG). (The representative result from three replicates are shown). (**b**) The molecular weight of GFP-N149DiZHSeC is measured by ESI-MS as 27944 Da (calculated 27941 Da). (The representative result from two replicates is shown). (**c**) DiZHSeC and DiZPK show similar photocrosslinking efficiency on the model protein HdeA. *E. coli* cells expressing the periplasm-residing HdeA-V58DiZPK or HdeA-V58DiZHSeC protein (carrying a C-terminal His tag) were incubated at pH 2.3 for 30 min followed by ultraviolet irradiation at 365 nm. Cell extracts are separated by the SDS–PAGE gel and analysed by anti-His immunoblotting. The HdeA monomer is marked with a black arrow and the crosslinked complexes are marked with a black brace. (The representative result from three replicates is shown).

After verifying the chemical properties of DiZHSeC, we started by testing the site-specific incorporation of DiZHSeC into model proteins. The DiZPK-recognizing PylRS mutant (L274A, C313S and Y349F)-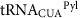 pair showed a similar amber suppression efficiency in the presence of DiZHSeC or DiZPK when an in-frame amber mutation site was introduced at residue N149 in green fluorescent protein (Fig. 2a). We expressed and purified the resulting protein GFP-N149DiZHSeC in *E. coli* and verified its molecular weight by electrospray ionization (ESI)-MS, which confirmed the incorporation specificity and fidelity of DiZHSeC (Fig. 2b). Furthermore, we incorporated DiZHSeC at residue V58 in an *E. coli* acid stress chaperone HdeA and found that the resulting protein HdeA-V58DiZHSeC exhibited similar efficiency in photo-affinity capturing of client proteins in *E. coli* periplasm as that of HdeA-V58DiZPK bearing the DiZPK photocrosslinker (Fig. 2c).

### Verification of the MS-label by fluorogenic labelling

**Figure 3:**
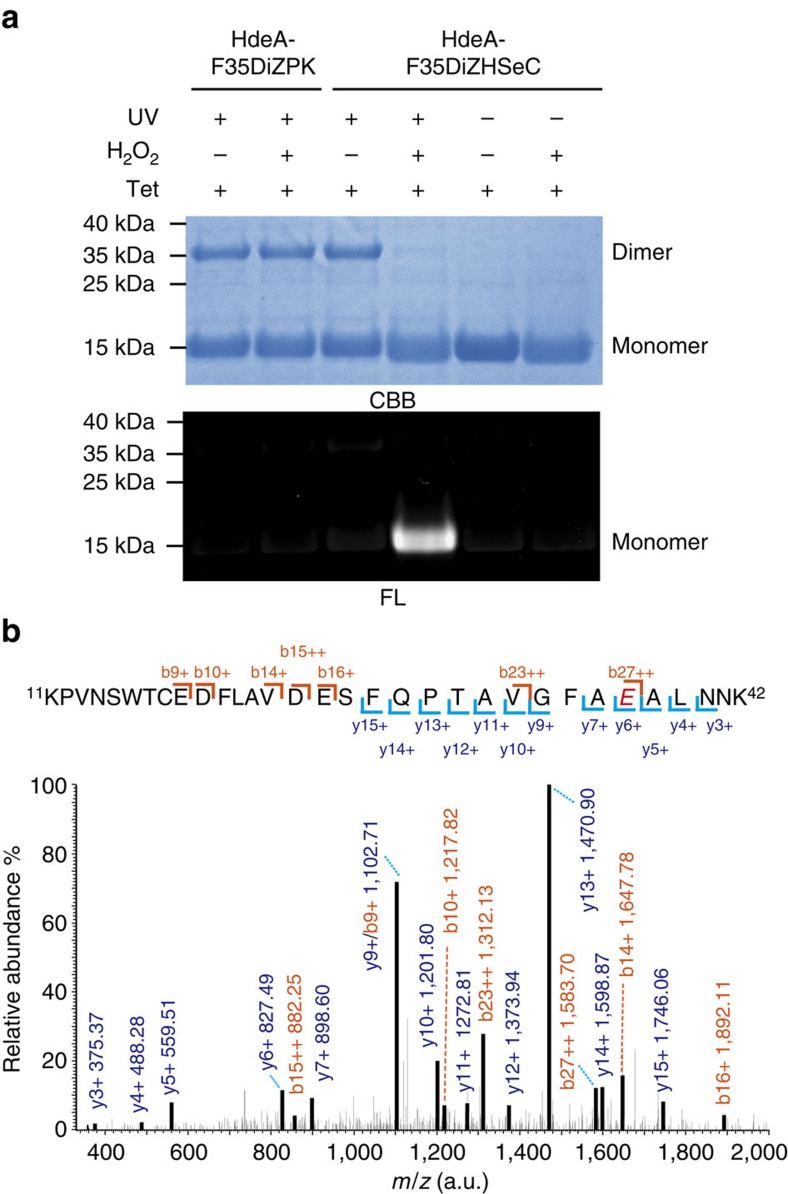
Verification and identification of the *in situ* generated MS-label from DiZHSeC. (**a**) Specific fluorogenic labelling of the *in situ* generated NPAA MS-label on prey proteins after photocrosslinking and oxidative cleavage of DiZHSeC via a crosslinked HdeA dimer model. CBB, coomassie brilliant blue staining; FL, fluorescent gel. (The representative result from three replicates is shown). (**b**) MS/MS spectra of a crosslinking peptide with the NPAA modification assigned to residue E37 (coloured in red) on HdeA. (The representative result from three replicates is shown).

We then examined the capability of DiZHSeC for H_2_O_2_-mediated cleavage and *in situ* generation of the NPAA MS-label. After photo-crosslinking and oxidative cleavage, DiZHSeC is expected to generate an NPAA moiety (C_8_H_13_NO) on the prey proteins containing an acrylamide group that can be detected by a tetrazole-containing fluorogenic probe (Tet) via ultraviolet -mediated cycloaddition reaction (‘photo-click' reaction, Supplementary Fig. 3; ref. 27). Using HdeA as a model protein, we first examined the stability of the *in situ* generated NPAA moiety. When incorporated at residue F35 on HdeA's dimer interface, both DiZHSeC and DiZPK were able to photo-capture the other interacting monomer at pH 7. The crosslinked HdeA dimers, bearing either DiZHSeC or the non-cleavable DiZPK as a control, were then subjected to oxidative cleavage followed by ultraviolet-mediated conjugation with the Tet probe. As expected, only the DiZHSeC-crosslinked HdeA dimer can be efficiently cleaved by H_2_O_2_ and subsequently fluorogenic-labelled by Tet, indicating that the H_2_O_2_ treatment generated a stable acrylamide group *in situ* (Fig. 3a). In contrast, the DiZPK-crosslinked HdeA dimer did not undergo oxidative cleavage to generate the NPAA moiety and thus remained undetectable by in-gel fluorescence. Furthermore, the non-crosslinked HdeA-F35DiZHSeC protein alone was also not labelled by Tet after H_2_O_2_ treatment (Fig. 3a). Taken together, these results confirm that our DiZHSeC photocrosslinker is not only able to efficiently photo-capture interacting protein partners, but can also undergo the cleavage-mediated generation of a stable NPAA chemical label on prey proteins.

### Identification of the MS-label by mass spectrometry

To test if the NPAA moiety could be utilized as a MS-label for target identification by mass spectrometry, we used the HdeA-F35DiZHSeC monomer to photocrosslink with the WT-HdeA monomer at pH 7 (Supplementary Fig. 4). The crosslinked dimer was then subjected to in-gel oxidative cleavage, trypsin digestion and liquid chromatography–tandem-mass spectrometry (LC–MS/MS) analysis on a Thermo Velos-Elite Orbitrap mass spectrometer. Searching the MS/MS data with Mascot identified an adduct peptide (^11^KPVNSWTCEDFLAVDESFQPTAVGFA***E***ALNNK^42^) with the MS-label (C_8_H_13_NO) modification (Fig. 3b). The monoisotopic mass of this adducted peptide was observed as 3722.8006 Da (calculated as 3722.7927 Da, −2.12 p.p.m.), equal to sum of the mass of NPAA (139.0997 Da) plus the mass of the unmodified peptide (3583.6929 Da). The modification site was unambiguously assigned to E37 on HdeA based on the MS/MS spectra (Fig. 3b). As a control, the parallel preparation of the photocosslinked HdeA-F35DiZPK/WT-HdeA complex did not yield any NPAA modified peptides in LC–MS/MS analysis. When the non-cleavable photocrosslinker was used, the crosslinking peptides in the prey proteins were difficult to identify by commonly used software because they remained attached with the peptides in the bait protein. In contrast, our DiZHSeC probe enabled *in situ* transfer of the stable MS-label on the prey peptides upon cleavage-mediated prey–bait separation, which can be readily identified by mass spectrometry.

### Development of IMAPP

**Figure 4:**
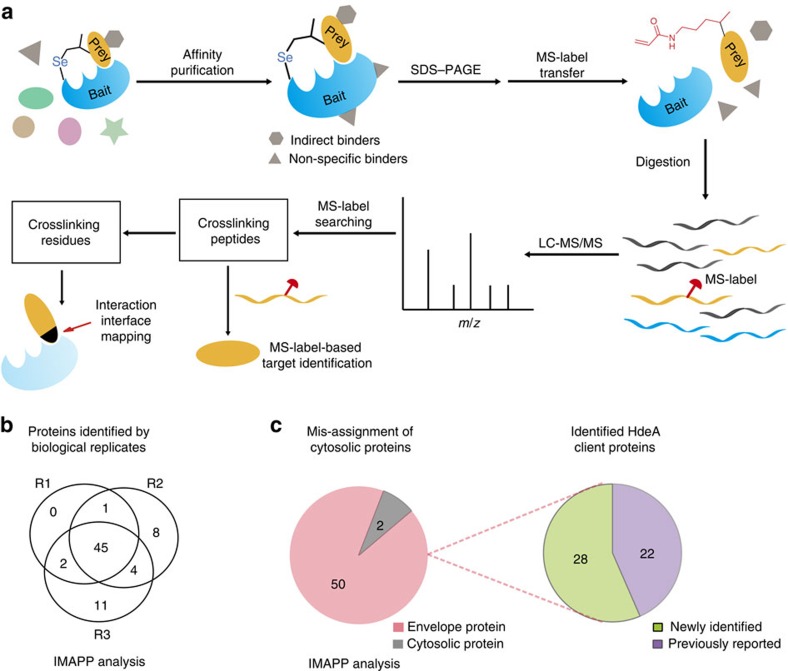
IMAPP-based profiling of intracellular protein–protein interactions using DiZHSeC. (**a**) A general workflow for IMAPP strategy. (**b**) Venn diagrams illustrating the number of HdeA client proteins identified in three biological replicates by IMAPP analysis. (**c**) The 52 proteins identified in at least two of the three replicates in IMAPP analysis. Left: percentage of mis-assignment of cytosolic proteins as HdeA client proteins. Envelope proteins are coloured in pink and cytosolic proteins are coloured in grey. Right: The 50 assigned HdeA client proteins include 22 previously reported clients (coloured in purple) and 28 newly identified clients (coloured in green), respectively. IMAPP analysis: a protein was assigned as a ‘hit' when the Mascot search identifies at least two unique peptides, including one crosslinking peptide with MS-label modification and another non-crosslinking peptide without MS-label.

Next, we utilized DiZHSeC to develop a MS-label assisted ‘IMAPP' strategy to directly capture and identify protein−protein interactions within living cells (Fig. 4a). In brief, the DiZHSeC-incorporated bait protein was expressed in cells and its native interactions with ‘prey' proteins were photo-captured under living conditions. The photocosslinked prey–bait complexes were enriched by affinity purification using an epitope tag on the bait protein, separated by SDS-PAGE gel and then subjected to in-gel H_2_O_2_-mediated oxidative cleavage and trypsin digestion. The digested peptides were analysed by LC–MS/MS, and the identity of the prey proteins as well as the crosslinking sites were assigned by searching for the specific MS-label (C_8_H_13_NO) modification on the peptides (Fig. 4a). Because DiZHSeC was installed within the specific interaction region on the bait protein, only the specific interaction partners can be photo-captured with an *in situ* generated MS-label upon cleavage. Therefore, by only focusing on peptides with a uniform MS-label modification after the IMAPP strategy, the crosslinking peptides can be readily distinguished from the remaining non-specific contaminants even after affinity purification^17^, which significantly increase the confidence for identifying the genuine pool of prey proteins.

### Identification of HdeA client proteins using IMAPP

As an essential acid stress chaperone in preserving the periplasmic proteostasis of enteric pathogens under acid stress (for example, pH 1–3 in human stomach), HdeA is able to bind a broad range of acid-susceptible client proteins to prevent them from aggregation^28^,^29^. Our previous efforts in identifying HdeA-binding partners at acidic conditions were hampered by false target identification due to non-specific protein binding and indirect protein interactions^14^,^19^. Therefore, we decide to apply the IMAPP strategy to re-evaluate this important protein−protein interaction network during bacterial acid resistance. The HdeA variants carrying DiZHSeC at different positions were confirmed to exhibit similar chaperone activity as WT-HdeA (Supplementary Fig. 5). *E. coli* cells expressing HdeA-V58DiZHSeC were treated at pH 2.3 for 30 min, irradiated with 365 nm ultraviolet light for 15 min and then subjected to the IMAPP strategy (Supplementary Fig. 6). During the traditional analysis without accounting for MS-label, a hit was assigned when the Mascot search identified at least two unique peptides from a given protein. A total of 967 hits were identified with 767 proteins appearing in at least two of three replicates, among which 402 (52% of the total) are cytosolic proteins (Supplementary Fig. 7). Because HdeA is a periplasmic chaperone that is expected to only interact with the envelope proteins, these cytosolic proteins are obviously false-positive hits, most likely originating from the affinity purification process. In contrast, when using IMAPP analysis that take the MS-label as a further criteria, a hit is assigned only when Mascot search identifies at least two unique peptides from a given protein, including one crosslinking peptide with the MS-label modification and another non-crosslinking peptide without MS-label modification. A total of 71 proteins were identified under this condition from *E. coli* K12 proteome, with 52 proteins identified in at least two of three replicates (Fig. 4b). These 52 proteins were all covered by the aforementioned 767 proteins identified by the traditional analysis. No hits containing MS-label were found in the control groups that were prepared either without ultraviolet irradiation or with WT-HdeA as the bait protein for ultraviolet irradiation (Supplementary Fig. 6). Among the 52 IMAPP hits, 50 (96%) are bacterial ‘envelope' proteins (located in periplasm as well as outer and inner membrane) while only 2 (4%) are cytosolic proteins (Fig. 4c). Therefore, in comparison with traditional analysis, the false-positive rate can be dramatically decreased with our IMAPP strategy. In addition, by focusing on the MS-label modified peptides, IMAPP strategy avoids the tedious peptide comparison between the experimental and control groups.

It is worth mentioning that the 50 envelope proteins identified by IMAPP strategy are not simply a reflection of protein abundance present in the periplasmic extract (Supplementary Table 1). This data indicates that HdeA indeed has its own client specificity when protecting clients from acid stress, with the underlying mechanism remaining elusive. The information regarding these 50 envelope clients as well as the photo-captured peptides carrying the MS-label are listed in Supplementary Tables 2 and 3. Twenty-two clients in this list have been reported before, including DegP and SurA, two essential periplasmic protein quality control (PQC) factors that have been previously shown to assist HdeA-mediated client proteins refolding during acid recovery (Fig. 4c; refs 14, 19). Interestingly, in the remaining 28 newly discovered HdeA client proteins we have identified several additional periplasmic PQC factors such as Tsp (a tail-specific protease), DsbA (a disulfide oxidoreductase) and YfgC (beta-barrel assembly-enhancing protease)^30^, and together with DegP and SurA, it suggests that a network of PQC factors may be protected by HdeA within *E. coli* periplasm under acid stress. Other newly discovered HdeA clients by our IMAPP method include inner membrane lipoproteins such as YfhM (α-macroglobulin), transport proteins such as YtfQ (ABC transporter periplasmic-binding protein) as well as other functional proteins such as Ecotin (a serine protease inhibitor). To further validate these MS results, we randomly chose four candidates (Tsp, BglX, DsbA, AspG2) and unambiguously detected their interaction with HdeA at pH 2 by fluorescence anisotropy (Supplementary Fig. 8), which confirmed the reliability of our IMAPP strategy for profiling intracellular protein–protein interactions.

Finally, as a further control, we applied the IMAPP strategy on the DiZHSeC-bearing HdeA at neutral pH, under which condition HdeA only binds to its dimer partner but not the client proteins. Photocrosslinking of the HdeA variant containing DiZHSeC at residue F35 on its dimer interface (HdeA-F35DiZHSeC) at pH 7 only yielded three candidate proteins, with HdeA itself being the dominant hit (Supplementary Fig. 9). Taken together, our IMAPP strategy offers a straightforward approach for highly efficient and confident identification of intracellular protein–protein interaction partners.

### Mapping HdeA dimer interface using IMAPP

**Figure 5:**
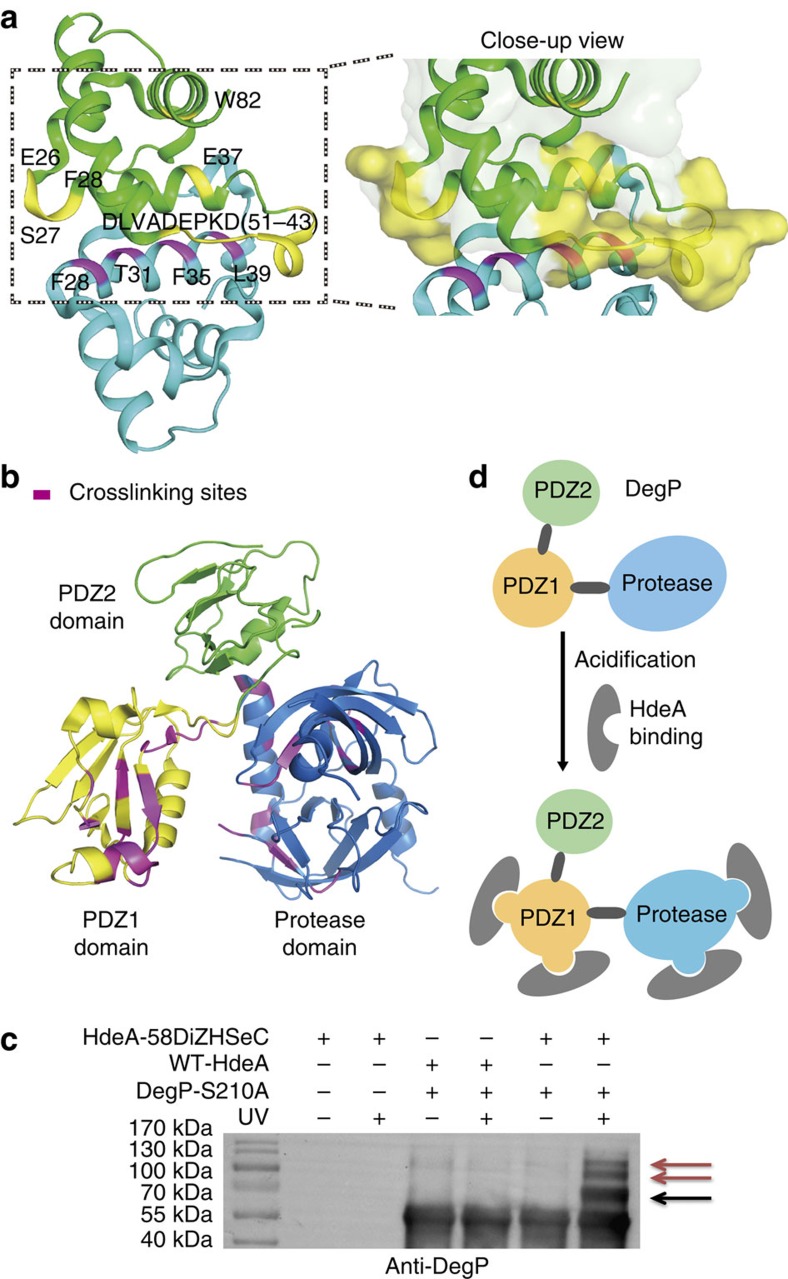
Mapping protein–protein interaction interface using IMAPP. (**a**) Mapping of HdeA dimer interface using the IMAPP strategy. DiZHSeC was incorporated at different sites (F28, T31, F35 and L39), respectively, on HdeA to photocrosslink with WT-HdeA at pH 7. The incorporation sites of DiZHSeC (coloured in magentas) and the crosslinking sites (coloured in yellow) are displayed on the crystal structure of HdeA (PDB: 1DJ8; ref. 28). The bait HdeA is coloured in cyan and the crosslinked HdeA is coloured in green. Close-up view of the crosslinking interface is shown in the left. Crosslinked HdeA monomer is shown as a surface representation with crosslinking residues coloured in yellow and the other residues coloured in green. (The representative result from three replicates is shown). (**b**) Interaction interface of HdeA-DegP mapping through the photocrosslinking sites identified by IMAPP strategy. The crystal structure of DegP (PDB: 3MH4; ref. 34) contains a protease domain (coloured in blue) and two PDZ domains (colour in yellow and green, respectively). Based on the integrated crosslinking sites identified by IMAPP (coloured in magenta), the HdeA–DegP interface can be mapped to the protease domain and the PDZ1 domain (The representative result from two replicates is shown). (**c**) Multiple HdeAs may bind to a single DegP molecule under acidic conditions. HdeA-V58DiZHSeC or WT-HdeA was used to photocrosslink with DegP-S210A and the crosslinked complexes were analysed by immunoblot. The crosslinked complex with a ratio of 1:1(HdeA/DegP) binding is marked with a black arrow. Crosslinked complexes with higher HdeA/DegP binding stoichiometries are marked with red arrows. (The representative result from three replicates is shown). (**d**) A proposed model illustrating that HdeA may interact with different regions on DegP while multiple HdeA chaperone molecules may also simultaneously bind to the same DegP molecule under acid stress.

Identification of the crosslinking sites is highly valuable for studying protein interaction interface^4^,^12^. However, traditional crosslinking experiments often require intensive software development to deconvolute the complicated MS spectra in order to identify such crosslinking peptides^31^,^32^. In contrast, this technical challenge can be addressed by using our photocrosslinker DiZHSeC. We postulated that our IMAPP strategy could be further utilized to map the interface of protein–protein interactions via these assigned crosslinking sites. For proof-of-concept, we first mapped HdeA dimer interface by photocrosslinking between the DiZHSeC-containing HdeA monomer and WT-HdeA monomer at pH 7. The crosslinking sites between WT-HdeA and HdeA-F35DiZHSeC monomer were identified by IMAPP as residues E37, D43, K44, DAVLD (47–51) and W82 (Supplementary Table 4 and Supplementary Fig. 10c). Based on the crystal structure of HdeA dimer (PDB: 1DJ8)^28^, the distances between F35 and these identified crosslinking residues (measured from C(α) of F35 to the closest carbon atom of each of the identified crosslinking residues) all fall within 14 Å which is the crosslinking range limit of DiZHSeC as measured from its C(α) to the photo-affinity center (Supplementary Fig. 11 and Supplementary Table 4). We further incorporated DiZHSeC at three additional sites (F28, T31 and L39) on HdeA dimer interface and found that their respective crosslinking sites were all located within the crosslinking range of DiZHSeC (Supplementary Fig. 12, Supplementary Fig. 10a,b,d and Supplementary Table 4). The incorporation of DiZHSeC into HdeA on the mentioned residues did not alter its structure as verified by circular dichroism spectroscopy (Supplementary Fig. 13). Integration of these crosslinking sites allowed us to map the HdeA dimer interface, which is highly consistent with that illustrated by the crystal structure (Fig. 5a). Traditionally, comprehensive mapping of the protein–protein interaction interface using site-specific crosslinkers requires genetic insertion of the photo-affinity UAA on both bait and prey proteins^11^,^12^,^13^. In contrast, such efforts can now be simplified by our IMAPP strategy as incorporation of the DiZHSeC probe at the bait side alone is sufficient to reveal the interaction interface on both sides.

### Probing the interaction dynamic change using IMAPP

In addition to static protein–protein interactions, we expected that our IMAPP strategy could also be applied to probe dynamic conformational changes that frequently occur at protein–protein interaction interfaces. Because HdeA is known to display pH-dependent conformational change^28^,^29^,^33^, we performed the IMAPP experiment at multiple pH conditions in order to capture such dynamic changes at the HdeA dimer interface (Supplementary Fig. 14). Upon acidification (pH<3), HdeA-F35DiZHSeC failed to crosslink with W82 in the peptide ‘^78^VKGEWDK^84^' located at the C-terminus of HdeA and this result is consistent with a structural model in which acid triggers the opening of the C terminal region of HdeA^33^. In addition, the original crosslinking site E37 on the peptide ‘^11^KPVNSWTCEDFLAVDESFQPTAVGFAEALNNK^42^' at pH 7 became spread to more residue positions within the peptide, indicating a potential order-to-disorder transition within this region when the environmental pH drops from 7 to 2 (Supplementary Fig. 15).

### Mapping HdeA–DegP interaction interface using IMAPP

Furthermore, we directly mapped a previously unknown protein–protein interaction interface using IMAPP. DegP is an essential PQC factor with dual protease and chaperone functions, and it contains one protease domain and two PDZ domains (PDZ1 and PDZ2; refs 14, 34). Our previous photocrosslinking experiments using DiZPK did not yield any information regarding the HdeA–DegP interaction interface on DegP side. According to our previous study, HdeA mainly interacts with its client proteins through two hydrophobic regions (Supplementary Fig. 16a; ref. 14^14^). We thus incorporated DiZHSeC at residues T31, L39, V49, V58 (within these two hydrophobic regions) or residue A6 (in its N-terminal hydrophilic region) as a control, and performed photocrosslinking between these HdeA variants and DegP-S210A (the catalytic dead mutant of DegP to avoid self-proteolysis) under acidic conditions. In agreement with the previous study, the crosslinking results showed that HdeA interacts with DegP mainly through its two hydrophobic regions (Supplementary Fig. 16a). The crosslinked complexes were further subjected to IMAPP strategy to analyse the crosslinking sites on DegP-S210A. The results indicated that HdeA directly interacted with DegP's protease domain and PDZ1 domain, but not the PDZ2 domain (Supplementary Fig. 16b–g, and Fig. 5b). We further applied the IMAPP strategy in living *E. coli* cells expressing HdeA-V58DiZHSeC and searched for the MS-label modified DegP peptides. Consistent with our *in vitro* data, the *in vivo* experiments also identified several crosslinking sites within the protease domain (Supplementary Table 3), suggesting that binding of HdeA at this region could be essential for protecting DegP from acid-induced damage. Furthermore, crosslinked complexes with the molecular weight higher than the 1:1 ratio complex (HdeA:DegP) were observed (Supplementary Fig. 17, and Fig. 5c). This result, together with the fact that the crosslinking peptides and sites located at multiple distinct regions on DegP, suggests that HdeA may interact with different regions on DegP while multiple HdeA chaperone molecules may also simultaneously bind to the same DegP molecule under acid stress (Fig. 5d). The structural basis for this intriguing domain-recognition specificity and the binding stoichiometry of HdeA towards DegP remains to be further verified.

### Expanding IMAPP to mammalian cells

Finally, as the Pyl-based genetic code expansion system has been widely adapted for various prokaryotic and eukaryotic systems^26^,^35^, we expect that our IMAPP strategy can be applied to identify protein–protein interactions in mammalian cells. As a proof-of-concept, we chose to study the interaction between RhoA and RTKN as well as their binding interface. RhoA is a Rho family small GTPase that has important roles in regulating actin cytoskeleton as well as cell adhesion^36^. As an effector protein for RhoA, RTKN can specifically interact with activated RhoA through its Rho-binding domain 1 (RH1 domain), which can inhibit the GTPase activity of RhoA^37^,^38^. To illustrate this specific and important interaction under living conditions, we chose to incorporate DiZHSeC at residue L45 within the RH1 domain of RTKN in an attempt to capture RhoA by photocrosslinking. HEK 293T cells expressing WT-RTKN or DiZHSeC-bearing RTKN with the constitutively active form of RhoA^G14V^ were subjected to ultraviolet irradiation and then analysed by immunoblotting^39^,^40^. To our delight, RTKN-L45DiZHSeC can efficiently capture its RhoA partner (Supplementary Fig. 18a), and the subsequent IMAPP analysis identified RhoA as the dominant hit (∼91% of relative abundance as calculated by NASF value) (Supplementary Fig. 18b). Furthermore, the crosslinking sites on RhoA were identified as residues Y66/D67/R68 that are located in the expected region known to be involved for contacting RhoA effector proteins such as PKN (Supplementary Fig. 18c; refs 41,42). This observation is also in agreement with the previous report showing that RTKN may interact with RohA through a similar binding interface as that on PKN^38^. Taken together, these data further demonstrated the capability of our IMAPP strategy for high-confidence identification of protein–protein interaction as well as simultaneous mapping of binding interface in various living species.

## Discussion

Coupling chemical crosslinking or photocrosslinking reagents with mass spectrometry has become a valuable tool for identifying protein–protein interactions, particularly under living conditions^1^,^2^,^3^,^4^. Chemical crosslinking is a well-developed strategy for identification of interaction partners as well as interaction interface, especially in the unbiased global proteomics study without the need for protein engineering^2^,^32^. However, such strategies suffer from the following limitations, especially in living systems: (i) the restriction to only a small set of chemically reactive amino acids, which may miss some interaction information when such residues are lacking; (ii) low efficiency under extreme conditions, for example, certain chemical crosslinkers such as disuccinimidyl suberate are not compatible with acidic conditions below pH 4.5; (iii) complicated crosslinking results that often include both intermolecular and intramolecular complexes, which requires intensive software development to decipher^2^,^3^,^31^,^32^.

By bearing a photo-labile moiety that can be site-specifically incorporated into proteins of interest, the genetically encoded photocrosslinkers allow facile capture of transient protein–protein interactions with high spatiotemporal resolution in living cells, which can largely overcome the aforementioned limitations for chemical crosslinking. Nevertheless, the downstream process after photocrosslinking still falls into the regular affinity purification procedure that suffers from false-positive identifications due to high contamination backgrounds. Laborious optimization of purification procedures including the washing condition, the design of tandem purification protocol, as well as tedious comparison with the control groups are usually needed in order to remove these contaminants, and the effectiveness of such efforts are highly variable in different cases^17^,^43^,^44^,^45^. Moreover, certain contaminants may still be difficult to be effectively removed, especially for those sticky indirect binders^46^. In addition, the information regarding protein interaction interface is particularly lacking, mainly due to the difficulties in deciphering crosslinking peptides and sites with traditional photocrosslinkers.

To overcome these limitations, we have developed a selenium-based, genetically encoded photo-affinity probe DiZHSeC that contains a transferable MS-label, allowing simultaneous identification of protein–protein interactions and mapping the corresponding contacting interfaces under living conditions. Taking the *in situ* transferred MS-label as an internal criterion, IMAPP allows high-confidence target identification from complicated proteome backgrounds without tedious optimization of purification procedure or time-consuming comparison with the control groups. Although the utilization of MS-label as the searching criterion may miss certain hits due to the relatively low abundance of modified peptides in the context of non-modified peptides during MS analysis, we expect that such a limitation can be addressed by introducing an affinity tag to the current photocrosslinker system that enables the enrichment of the MS-label-modified peptides.

Notably, the exact crosslinking sites can be readily identified from the MS data through IMAPP analysis, permitting simultaneous illustration of protein–protein interaction interface. Albeit being a relatively low-resolution method in mapping such interfaces, this approach is highly valuable in providing dynamic and real-time structural information regarding protein interaction networks under living conditions. To our knowledge, there are currently no genetically encoded photocrosslinking probes that are able to achieve these multiple challenging goals simultaneously. In particular, as exemplified by our study on the acid chaperone HdeA with its client DegP here, this strategy is especially well-suited for studying protein–protein interactions that involve conditionally or intrinsically disordered proteins which remain almost inaccessible by other conventional structural methods such as X-ray crystallography.

## Methods

### Plasmids construction

The plasmid *pSupAR-MbPylRS* encoding the mutant MbPylRS and its cognitive tRNA_CUA_^Pyl^ (MbPylRS recognizes and transfers the UAA to tRNA_CUA_^Pyl^, which inserts the UAA into the in-frame amber code site on target genes) for DiZHSeC in *E.coli* cells were described in the previous literature^14^. The plasmids encoding the GFP, HdeA, DegP-S210A or their mutant variants (carrying a C-terminal His-tag) and the plasmid encoding the WT-HdeA carrying no tag on its C-terminus were described in previous literature^14^,^19^. The plasmid *pBAD-HdeA-A6TAG-His*_*6*_ and *pBAD-HdeA-F28TAG-His*_*6*_, *pBAD-HdeA-T31TAG-His*_*6*_, *pBAD-HdeA-L39TAG-His*_*6*_, *pBAD-HdeA-V49TAG-His*_*6*_ encoding the mutant HdeA (carrying a C-terminal His tag) were generated using site-directed mutation on the plasmid *pBAD-HdeA-His*_*6*_.

*pET28a-Tsp-His_6_*. The Tsp sequence was amplified from the genome of *E. coli (DH10B)* by PCR with primers 5′-CATGCCATGGCGAACATGTTTTTTAG (5′ primer), and 3′-CCGCTCGAGCTTGACGGGAGCGGGTTGTTC (3′ primer). Then, the insert was cloned into *Nco*I/*Xho*I sites on a *pET-28a* vector to produce the final plasmid *pET28a-Tsp-His*_*6*_.

*pBAD-BglX-His_6_*. The BglX sequence was amplified from the genome of *E. coli (DH10B)* by PCR with primers 5′-CCGCTCGAGCAAATGGCTATGTTCAG (5′ primer), and 3′-CGGAATTCGTCAGCAACTCAAACTC (3′ primer). Then, the insert was cloned into *Xho*I/*Eco*RI sites on a *pBAD-myc-His/A* vector to produce the final plasmid *pBAD-BglX-His*_*6*_.

*pBAD-AspG2-His_6_*. The AspG2 sequence was amplified from the genome of *E. coli (DH10B)* by PCR with primers 5′-CATGCCATGGACGAGTTTTTCAAAAAG (5′-primer) and 3′-CGGAATTCGTGTACTGATTGAAGATC (3′-primer). Then, the insert was cloned into *Nco*I/*Eco*RI sites on a *pBAD-myc-His/A* vector to produce the final plasmid *pBAD-AspG2-His*_*6*_.

*pBAD-DsbA-His_6_*. The DsbA sequence was amplified from the genome of *E. coli (DH10B)* by PCR with primers 5′-CATGCCATGGACAAAAAGATTTGGCTG (5′-primer) and 3′-CGGAATTCGTTTTTTTCTCGGACAG (3′-primer). Then, the insert was cloned into *Nco*I/*Eco*RI sites on a *pBAD-myc-His/A* vector to produce the final plasmid *pBAD-DsbA-His*_*6*._

The plasmid *pCMV-MbPylRS* encoding the mutant MbPylRS and its cognitive tRNA_CUA_^Pyl^ for DiZHSeC in mammalian cells were described in the previous literature^35^.The plasmid *pCMV-RTKN-flag* encoding the RTKN protein containing a flag tag on its C-terminus was a gift from Dr Guifang Jia group in Peking University. The plasmid *pCMV-L45TAG-RTKN-flag* were generated using site-directed mutation on the plasmid *pCMV-RTKN-flag*. Plasmid *pCMV-RhoA-myc* encoding the RhoA protein containing a myc tag on its C-terminus was purchased from Sino Biological Inc. (Beijing, China). Plasmid *pCMV-RhoA*^*G14V*^*-myc* encoding the constitutively active RhoA^G14V^ was generated using the site directed mutation on the plasmid *pCMV-RhoA-myc*.

### Reagents and equipments

Compounds used in the synthesis of DiZHSeC were purchased from J&K Scientific, Aladdin or Alanine. All chemicals used in this study were analytical grade or above. Primary antibody: anti-His antibody (ZSGB-bio, 1:2,000 diluted); anti-DegP antibody was raised in rabbits (1:20,000 diluted)^29^; anti-flag antibody (Sigma, 1:2,000 diluted); anti-myc antibody (ZSGB-bio, 1:2,000 diluted). Horseradish peroxidase-linked secondary antibodies were purchased from Cell Signaling Technology (1:5,000 diluted).

^1^H NMR and ^13^C NMR spectra were recorded on a Bruker-500 MHz NMR (AVANCE III). High resolution mass spectra (HRMS) were recorded on a Fourier Transform Ion Cyclotron Resonance Mass Spectrometer (APEX IV). NMR spectra and HRMS spectra of the compounds in this article are available in Supplementary Figs 19–24. Protein purifications were performed on an AKTA UPC 900 system (GE healthcare). Protein mass spectrometry (LC–MS) was performed on an ACQUITY UPLC I-Class SQD-2 (Waters) system with ESI. LC–MS/MS analysis of tryptic peptides was performed on an LTQ-Orbitrap-Elite mass spectrometer coupled with an Easy nLC 1,000 system (Thermo Scientific). Images of protein gels including coomassie SDS–PAGE gel, fluorescent gel and western blotting membrane were taken on ChemiDoc XRS (Bio-Rad). Full image of the blots and gels in the main paper are available in Supplementary Fig. 25. Circular dichroism spectroscopy measurement was performed on a J-815 CD-spectrometer (JASCO). Fluorescence anisotropy experiment and light scattering experiment were performed on a Cary Eclipse Fluorescence Spectrophotometer (Agilent Technologies).

### Characterization of newly synthesized compounds

The synthesis route for DiZHSeC is described in Supplementary Fig. 1. The method for synthesis is described in Supplementary Methods. For NMR analysis and HRMS analysis of the compounds in this article; see Supplementary Figs 19–24.

*Compound **2***. ^1^H NMR (500 MHz, CDCl_3_): *δ* 3.64 (t, *J*=6.6 Hz, 2H), 3.23–3.25 (m, 2H), 2.78 (t, *J*=6.6 Hz, 2H), 1.39–1.45 (m, 4H), 1.02 (s, 3H); ^13^C NMR (125 MHz, CDCl_3_): *δ* 170.05, 39.54, 39.08, 31.67, 27.71, 25.46, 24.12, 19.79; HRMS (*m*/*z*): [M+H]^+^ calcd for C_8_H_15_BrN_3_O, 248.03985; found 248.03903.

*Compound DiZHSeC*. ^1^H NMR (500 MHz, CD_3_OD/D_2_O/DCl): *δ* 4.14 (t, *J*=6.4 Hz, 1H), 3.18 (t, *J*=6.6 Hz, 2H), 2.85 (t, *J*=7.2 Hz, 2H), 2.75–2.79 (m, 2H), 2.67 (t, *J*=7.2 Hz, 2H), 2.25–2.35 (m, 2H), 1.40–1.42 (m, 4H), 1.00 (s, 3H); ^13^C NMR (CD_3_OD/D_2_O/DCl, 125 MHz): *δ* 173.54, 170.06, 52.43, 38.79, 36.33, 31.30, 31.13, 25.05, 23.40, 18.52, 17.97, 17.77; HRMS (*m*/*z*): [M+H]^+^ calcd for C_12_H_23_N_4_O_3_Se, 351.09354; found 351.09232.

### Expression of UAA-incorporated proteins in *E. coli*

Expression of UAA-incorporated proteins was carried out in *DH10B* cells co-transformed with plasmids carrying both the *Mb*PylRS mutant/tRNA_CUA_^Pyl^ pair and the target protein gene with an in-frame amber codon on the incorporation site. The overnight cultured bacterial cells harbouring these two plasmids were 1:100 diluted with fresh LB medium containing 50 mg l^−1^ ampicillin and 34 mg l^−1^ chloramphenicol. The bacteria were then grown at 37 °C to an OD_600_∼0.5 before DiZHSeC or DiZPK was added to a final concentration of 330 μM. After 30 min incubation, protein expression was induced by the addition of arabinose to give a final concentration of 0.2% and cells were harvested after 12 h expression at 30 °C.

### Protein purification

Five-hundred millilitre *E.coli* (*DH10B*) expressing the proteins carrying a C-terminal His-tag were harvested by centrifugation at 6,000 r.p.m. for 20 min before being suspended in lysis buffer (20 mM Tris-HCl, 500 mM NaCl, pH 7.4). Lysate after sonication was loaded onto a Ni-NTA column (Histrap 5 ml, GE healthcare), which was washed with 40 ml washing buffer (20 mM Tris-HCl, 500 mM NaCl, 40 mM imidazole, pH 7.4) and eluted with elution buffer (20 mM Tris-HCl, 500 mM NaCl, 250 mM imidazole, pH 7.4). The eluted protein was then desalted to 1 × PBS buffer (150 mM NaCl, 1.5 mM KH_2_PO_4_, 8 mM Na_2_HPO_4_, pH 7.4).

Five-hundred millilitre *E. coli (BL21)* cells expressing WT-HdeA protein carrying no tag on its C terminal was harvested by centrifugation at 6,000 r.p.m. for 20 min before being suspended in buffer A (20 mM Tris-HCl, pH 7.4). Lysate after sonication was loaded onto a HiTrap QFF column (5 ml, GE healthcare). The column was washed with one column volume of buffer A (20 mM Tris-HCl, pH 7.4) before being eluted with a 0–0.08 M sodium chloride salt gradient in buffer A. After being identified by SDS–PAGE, the fractions containing the WT-HdeA protein were desalted to buffer A and then loaded on a Mono Q 5/50 GL column (GE healthcare). The column was then washed with one column volume of buffer A before being eluted with a 0–0.08 M sodium chloride salt gradient in buffer A. After being identified by SDS–PAGE, the fractions containing the WT-HdeA protein were collected and desalted to 1 × PBS buffer (150 mM NaCl, 1.5 mM KH_2_PO_4_, 8 mM Na_2_HPO_4_, pH 7.4; ref. 29).

### ESI-MS analysis

LC–MS analysis of GFP-N149DiZHSeC was performed using a Waters ACQUITY UPLC I-Class SQD 2 mass spectrometer with ESI. In all 0.1% formic acid in H_2_O as buffer A and 0.1% formic acid in acetonitrile as buffer B were taken as the solvent system. LC separation for GFP-N149DiZHSeC was carried out with a BEH300 C4 Acquity column (1.7 μm, 2.1 × 100 mm), and positive mode was chosen for ESI-MS to analyse all samples. Total mass of the protein was calculated by MassLynx V4.1 software (Waters). Theoretical mass of the wild-type protein was calculated using PROTEIN CALCULATOR v3.3 (http://www.scripps.edu/~cdputnam/protcalc.html), and theoretical mass for the modified protein was adjusted manually.

### *In vivo* photocrosslinking of HdeA dimmers

*E.coli (DH10B)* cells expressing HdeA-F35DiZHSeC or HdeA-F35DiZPK (carrying a C-terminal His-tag) were harvested at 4,000 r.p.m. for 10 min. The supernatant was discarded, and the bacterial pellet was suspended in LB buffer at pH 7. Then the sample was irradiated by ultraviolet for 15 min with UVP CL-1000 ultraviolet Cross-Linker installed with 365 nm ultraviolet lamps (Hitachi F8T5/Black Light, 8-watt) at a distance of ∼5 cm (∼2 mw cm^−2^) on ice. The crosslinked dimer was purified according to the method mentioned above.

### Fluorogenic labelling

The *in vivo* crosslinked HdeA (carrying a C-terminal His-tag) dimers (pH 7) using DiZHSeC or DiZPK as the photocrosslinker (or the non-crosslinked bait protein HdeA-F35DiZHSeC, carrying a C-terminal His-tag) (1 mg ml^−1^) were denatured by 1% SDS and incubated with 8 mM H_2_O_2_ in PBS (pH 8.0) at 37 °C for 3 h. After removal of the remaining H_2_O_2_ by desalting with Micro Bio-spin 6 columns (BIO-RAD), the solution was incubated with 200 μM Tet probe upon ultraviolet irradiation for 5 min. Then the proteins were separated by the SDS–PAGE gel, and followed by coomassie blue staining or in-gel fluorescence analysis.

### *In vitro* photocrosslinking of protein complexes

*Photocrosslinking of HdeA-DiZHSeC/WT-HdeA heterodimer at pH 7*. A solution (160 μl) of 30 μM HdeA-DiZHSeC (carrying a C-terminal His-tag) and 30 μM WT-HdeA (carrying no tag) was first incubated at pH 2.0 for 30 min at 37 °C and then at 7.0 for another 30 min to promote heterodimer formation at neutral pH. The mixture was irradiated by ultraviolet for 15 min. The solution volume was concentrated to 40 μl using Amicon Ultra-0.5 Centrifugal Filter Devices (10 K) and the crosslinked complexes were separated by the SDS–PAGE gel followed by coomassie blue staining.

*Photocrosslinking of HdeA-F35DiZHSeC/WT-HdeA heterodimer at pH 2*. A solution (160 μl) of 30 μM HdeA-F35DiZHSeC (carrying a C-terminal His-tag) and 30 μM WT-HdeA (carrying no tag) was incubated at pH 2.0 for 30 min at 37 °C followed by ultraviolet irradiation for 15 min. The solution was recovered to pH 7 using 0.6 M NaOH and the solution volume was concentrated to 40 μl using Amicon Ultra-0.5 Centrifugal Filter Devices (10 K). Then the crosslinked complexes were separated by the SDS–PAGE gel followed by coomassie blue staining.

*Photocrosslinking of HdeA-DiZHSeC/DegP-S210A complex*. A solution (30 μl) of 50 μM HdeA-DiZHSeC (carrying a C-terminal His-tag) and 15 μM DegP-S210A was incubated at pH 2.0 for 30 min at 37 °C followed by ultraviolet irradiation for 15 min. Then the pH of the solution was recovered to 7 using 0.6 M NaOH. The crosslinked complex was separated by the SDS–PAGE gel followed by coomassie blue staining.

### *In vivo* photocrosslinking of HdeA with it client proteins

*E.coli (DH10B)* cells expressing HdeA-V58DiZHSeC were harvested at 4,000 r.p.m. for 10 min. The supernatant was discarded, and the bacterial pellet was suspended in LB buffer. The pH was adjusted to 2.3 using 5 M HCl, and the cells were incubated at 37 °C for 30 min, followed by ultraviolet irradiation for 15 min. The pH of the solution was then recovered to 7 using 5 M NaOH and the crosslinked prey–bait complexes were purified according to the method mentioned above.

### In-gel cleavage and digestion in IMAPP strategy

The crosslinked prey–bait complexes were separated by the SDS–PAGE gel, and the corresponding protein bands were excised and cut into pieces. The gel pieces were dehydrated in acetonitrile, then incubated in buffer I (10 mM dithiothreitol, 50 mM ammonium bicarbonate) at 56 °C for 30 min, and were further incubated in buffer II (55 mM iodoacetamide, 50 mM ammonium bicarbonate) at ambient temperature for 1 h in the dark before being dehydrated. The samples were then incubated in 8 mM H_2_O_2_ in PBS (pH 8.0) at 37 °C for 3 h, followed by washing with H_2_O for three times, and dehydrated again. Then the samples were in-gel digested with sequencing grade trypsin (5 ng μl^−1^ trypsin, 50 mM ammonium bicarbonate, pH 8.0) overnight at 37 °C. The resulting peptides were extracted twice with 5% formic acid/50% acetonitrile in water, and then vacuum-centrifuged to dryness.

### LC–MS/MS analysis in IMAPP strategy

To analyse the *in vivo* crosslinked protein complexes, the corresponding protein bands were excised and divided into six samples and, respectively, subjected to the in-gel cleavage and digestion procedure mentioned above. The extracted peptides were reconstituted in 0.2% formic acid, loaded onto a 100 μm × 2 cm pre-column and separated on a 75 μm × 20 cm capillary column both of which were packed in-house with 4 μm C18 bulk materials (InnosepBio, China). An Easy nLC 1000 system (Thermo Scientific, USA) was used to generate the following HPLC gradient: 7–35% B in 40 min, 35–75% B in 4 min, then held at 75% B for 20 min (A=0.1% formic acid in water, B=0.1% formic acid in acetonitrile). The eluted peptides were sprayed into an LTQ-Orbitrap-Elite mass spectrometer (Thermo Scientific, USA) equipped with a nano-ESI source. The mass spectrometer was operated in data-dependent mode with one MS scan in FT mode at a resolution of 30,000 followed by 10 CID (Collision Induced Dissociation) MS/MS scans in the ion trap for each cycle.

### Data analysis in IMAPP strategy

Raw data files produced in the Xcalibur software (Thermo Scientific) were transformed to mgf files through MSConvert and then searched with Mascot V.2.3.02 (Matrix Science) against SwissProt 57.15 (515,203 sequences; 181,334,896 residues) *E. coli* database (22,646 sequences). Searches were performed with a precursor mass tolerance set to 7 p.p.m., fragment mass tolerance set to 0.6 Da and a maximum number of missed cleavages set to 3. In addition to the regular cysteine carbamidomethylation (C_2_H_3_NO, 57.0215 Da), methionine oxidation (O, 15.9949 Da) and oxidation on a carbamidomethylated cysteine (C_2_H_3_NO_2_, 73.0164 Da), the following extra variable modifications were defined in the search in order to account for all possible scenarios of MS-label modifications: 1. MS-label modification on each of all 20 amino acids (C_8_H_13_NO, 139.0997 Da); 2. MS-label modification on methionine or cysteine+oxidation (C_8_H_13_NO_2_, 155.0946 Da). 3. MS-label modification on cysteine+carbamidomethylation (C_10_H_16_N_2_O_2_, 196.1212 Da) 4. MS-label modification on cysteine+carbamidomethylation+oxidation (C_10_H_16_N_2_O_3_, 212.1161 Da). The peptide ion score threshold was set according to the score distribution (*P* value<0.05, *E* value<0.05). For the *in vivo* crosslinking results, auto decoy search was applied to evaluate the false discovery rate (FDR) as 0.025 or below. For the traditional analysis without accounting for MS-label, a protein was assigned as a ‘hit' when the Mascot search identifies at least two unique peptides from it. For the IMAPP analysis, a protein was assigned as a ‘hit' when the Mascot search identifies at least two unique peptides, including one crosslinking peptide with MS-label modification and another non-crosslinked peptide without MS-label. The crosslinking sites on modified peptides were assigned based on the MS/MS spectra.

### *In vivo* photocrosslinking of DiZHSeC-incorporated RTKN

HEK 293T cells (HEK 293T/17, ATCC, CRL-11268) were co-transfected with plasmids *pCMV-MbPylRS*, *pCMV-RhoA*^*G14V*^*-myc* and *pCMV-TAG-RTKN-flag* by using Lipo2000 in DMEM containing fetal bovine serum (1%) and a DiZHSeC (200 μM) at a cell density of 80% for 6 h. After transfection, the medium was replaced by DMEM containing fetal bovine serum (10%) and DiZHSeC (1 mM) and the cells were allowed to grow for 12–16 h. Then the medium was change to Dulbecco's phosphate buffered saline (DPBS), and the cells were treated with ultraviolet irradiation for 15 min on the ice. After sonication in lysis buffer (50 mM HEPES, 500 mM KCl, 2 mM EDTA, 0.05% NP-40, 1% Triton-X100, pH 7.4), the lysate was incubated with anti-FLAG beads at 4 °C for 1 h. The beads were then washed with wash buffer (50 mM HEPES, 500 mM KCl, 2 mM EDTA, 0.05% NP-40, pH 7.4). The proteins were finally eluted with FLAG peptides followed by separation through SDS–PAGE gel and then subjected to IMAPP procedure mentioned above. The search was performed using Mascot V.2.3.02 (Matrix Science) against SwissProt 57.15 (515,203 sequences; 181,334,896 residues) *Homo sapiens* (human) database (20,266 sequences). The peptide ion score threshold was set according to the score distribution (*P* value<0.05, *E* value<0.05). A protein was assigned as a ‘hit' when the Mascot search identifies at least two unique peptides, including one crosslinking peptide with MS-label modification and another non-crosslinking peptide without MS-label.

### Data availability

The authors declare that the data supporting the findings of this study are available within the article and its Supplementary Information files. Any further relevant data concerning the techniques used in the paper are available from P.R.C. or C.W. on request.

## Additional information

**How to cite this article:** Yang, Y. *et al.* Genetically encoded protein photocrosslinker with a transferable mass spectrometry-identifiable label. *Nat. Commun.* 7:12299 doi: 10.1038/ncomms12299 (2016).

## Supplementary Material

Supplementary Information Supplementary Figures 1-25, Supplementary Tables 1-4, Supplementary Methods and Supplementary References.
