# Supplementary material for: Genetically encoded protein photocrosslinker with a transferable mass spectrometry-identifiable label
Source: Nat Commun. 2016 Jul 27;7:12299. doi: 10.1038/ncomms12299 (PMC4974458; doi:10.1038/ncomms12299)
Supplement: Supplementary Information — Supplementary Figures 1-25, Supplementary Tables 1-4, Supplementary Methods and Supplementary References. [file ncomms12299-s1.pdf]

Supplementary Figures

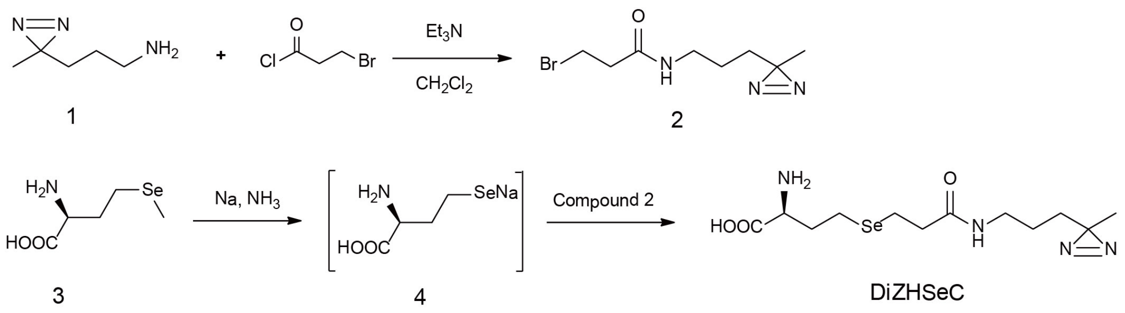

Supplementary Figure 1. The synthesis route of DiZHSeC.

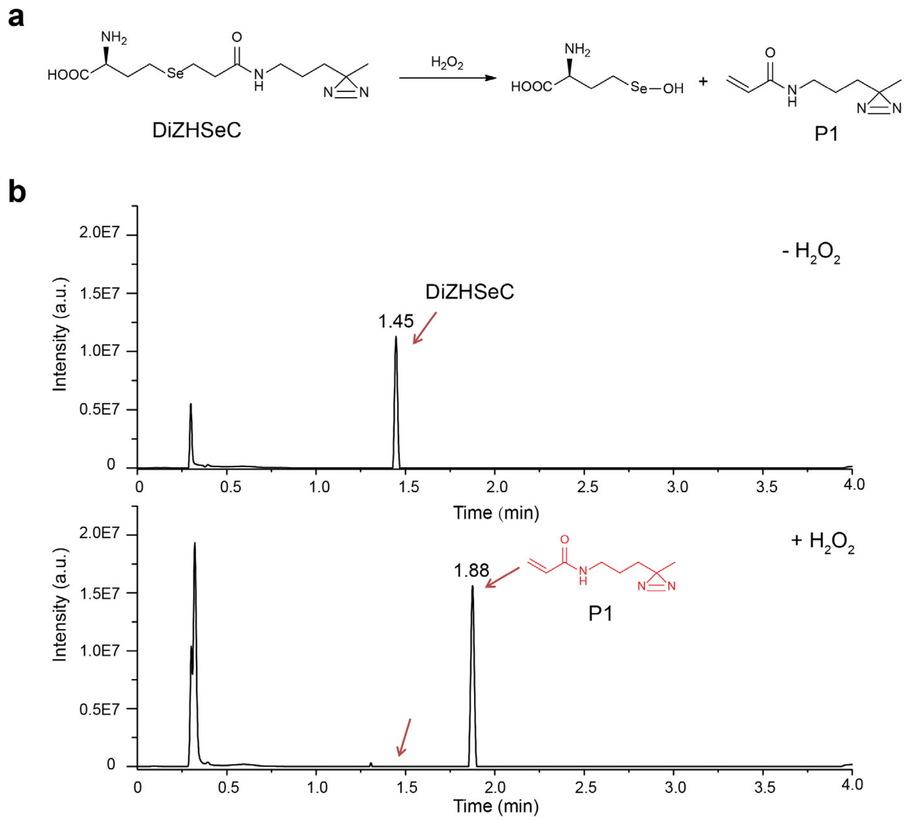

Supplementary Figure 2. Verification of the H_2O_2-mediated oxidative cleavage of DiZHSeC. (a) Oxidative cleavage of DiZHSeC by H_2O_2 is predicted to generate the product P1. **(b)** Verification of the cleavage reaction by UPLC-MS. 200 μM DiZHSeC was treated with or without 8 mM H_2O_2 for 1 h (pH 8.0, 37 °C) and then subjected to UPLC-MS analysis. Without H_2O_2 treatment (top), DiZHSeC elutes as a single peak (t = 1.45 min, m/z ($[M+Na]^+$) = 373.24 (calculated for 373.08)). Upon H_2O_2 treatment (bottom), the DiZHSeC peak disappears and a new peak appears instead corresponding to the cleaved product P1 (t = 1.88 min, m/z ($[M+Na]^+$) = 190.23 (calculated for 190.10)). a.u., arbitrary units. (The representative result from 3 replicates is shown).

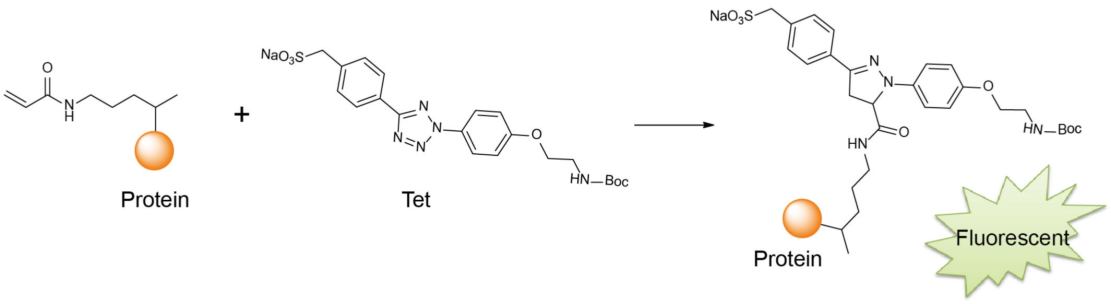

Supplementary Figure 3. Scheme for fluorogenic labeling of the NPAA moiety through UV-mediated cycloaddition reaction with the Tet probe[1].

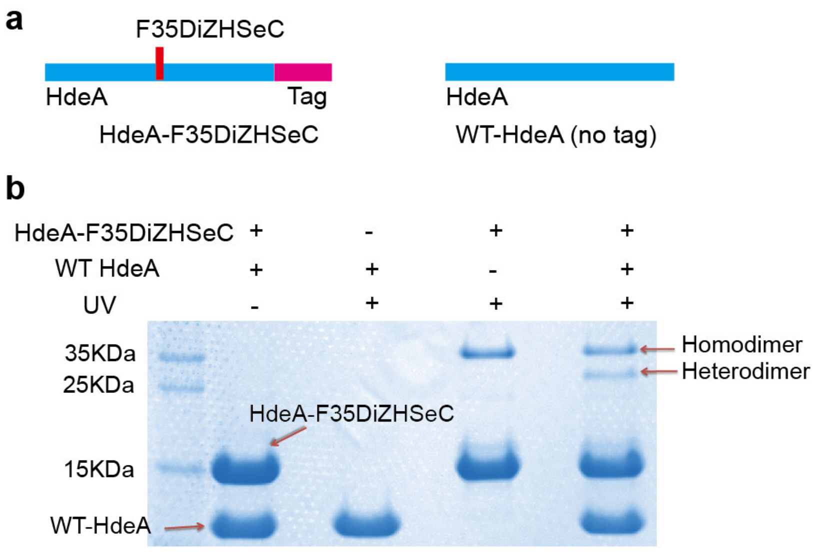

Supplementary Figure 4. Photocrosslinking of HdeA-F35DiZHSeC/WT-HdeA heterodimer. (a) HdeA-F35DiZHSeC was constructed with an epitope tag on its C-terminal, which results in an increased molecular weight compared to that of WT-HdeA without a tag. This allows HdeA-F35TAG and WT-HdeA to be separated and distinguished on a SDS-PAGE gel. **(b)** HdeA-F35DiZHSeC (served as the "bait") was used to photocrosslink with WT-HdeA (served as the "prey") to form the heterodimer complex. 30 μM HdeA-F35DiZHSeC and 30 μM WT-HdeA (or with either component) were incubated first at pH 2.0 for 30 min at 37 °C and then at 7.0 for another 30 min at 37 °C to promote heterodimer formation at neutral pH. The solution was treated with or without UV irradiation and subjected to the SDS-PAGE gel separation. Coomassie blue staining shows the formation of HdeA-F35DiZHSeC homodimer as well as the heterodimer complex of HdeA-F35DiZHSeC/WT-HdeA. (The representative result from 3 replicates is shown).

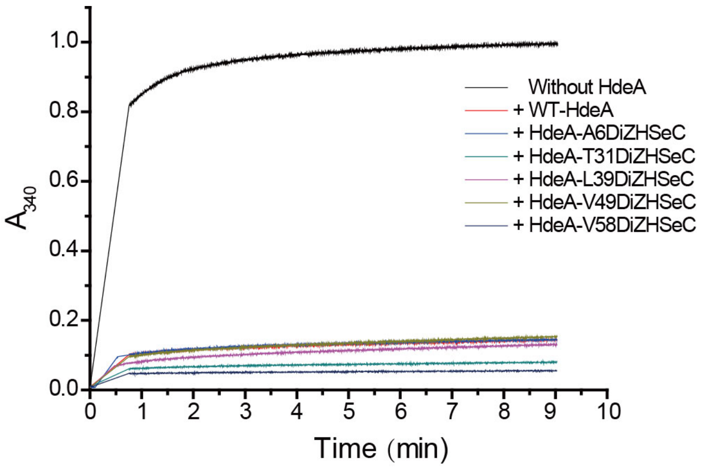

Supplementary Figure 5. HdeA-DiZHSeC variants showed similar activity as WT-HdeA. The amount of acid induced aggregation of SurA (2 μM) at pH 2 in the presence or absence of WT-HdeA or Mutant HdeA (8 μM) was monitored by following apparent changes in absorbance caused by light scattering at 340 nm. WT-HdeA and HdeA-DiZHSeC mutants show similar protection effects on acid induced aggregation of SurA. (The representative result from 3 replicates is shown).

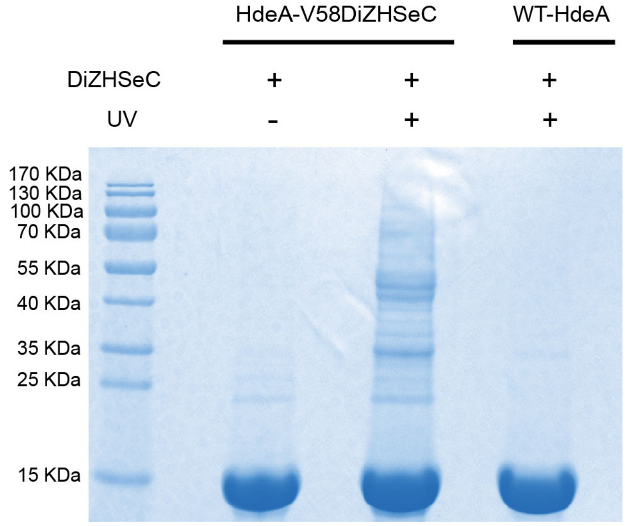

Supplementary Figure 6. Capture of the HdeA-binding proteins as preys via DiZHSeC-mediated photocrosslinking in living cells. HdeA is a periplasm-residing acid chaperon protecting its client proteins under acid condition. *E. coli* cells expressing either HdeA-V58DiZHSeC (carrying a C-terminal His-tag) or WT-HdeA (carrying a C-terminal His-tag) as the bait protein were incubated at pH 2.3 for 30 min followed by treatment with or without UV irradiation. The bait and prey-bait complexes were then purified through a Ni-NTA column and separated by the SDS-PAGE gel. Coomassie blue staining shows that only HdeA-V58DiZHSeC is able to photo-capture its client proteins to form prey-bait complexes with higher molecular weights. (The representative result from 3 replicates is shown).

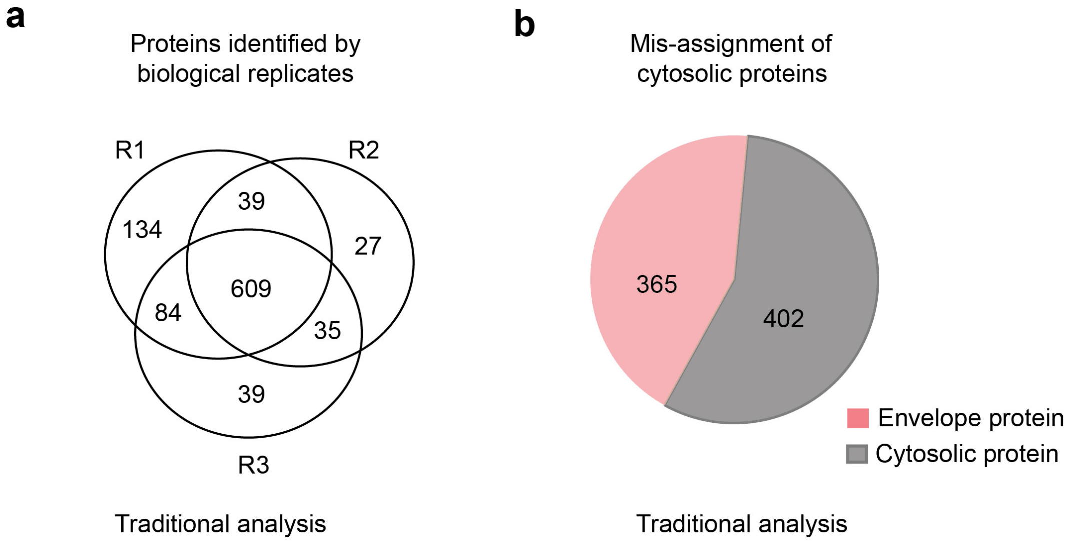

Supplementary Figure 7. Identification of HdeA client proteins using traditional analysis without accounting for MS-label. (a) Venn diagrams illustrating the number of HdeA client proteins identified in three biological replicates by traditional analysis without accounting for MS-label. **(b)** Mis-assignment of cytosolic proteins as HdeA client proteins in traditional analysis. The 767 proteins identified in at least two of three replicates using traditional analysis contains 365 envelope proteins (48%) and 402 cytosolic proteins (52%). Envelope proteins are colored in pink and cytosolic proteins are colored in grey.

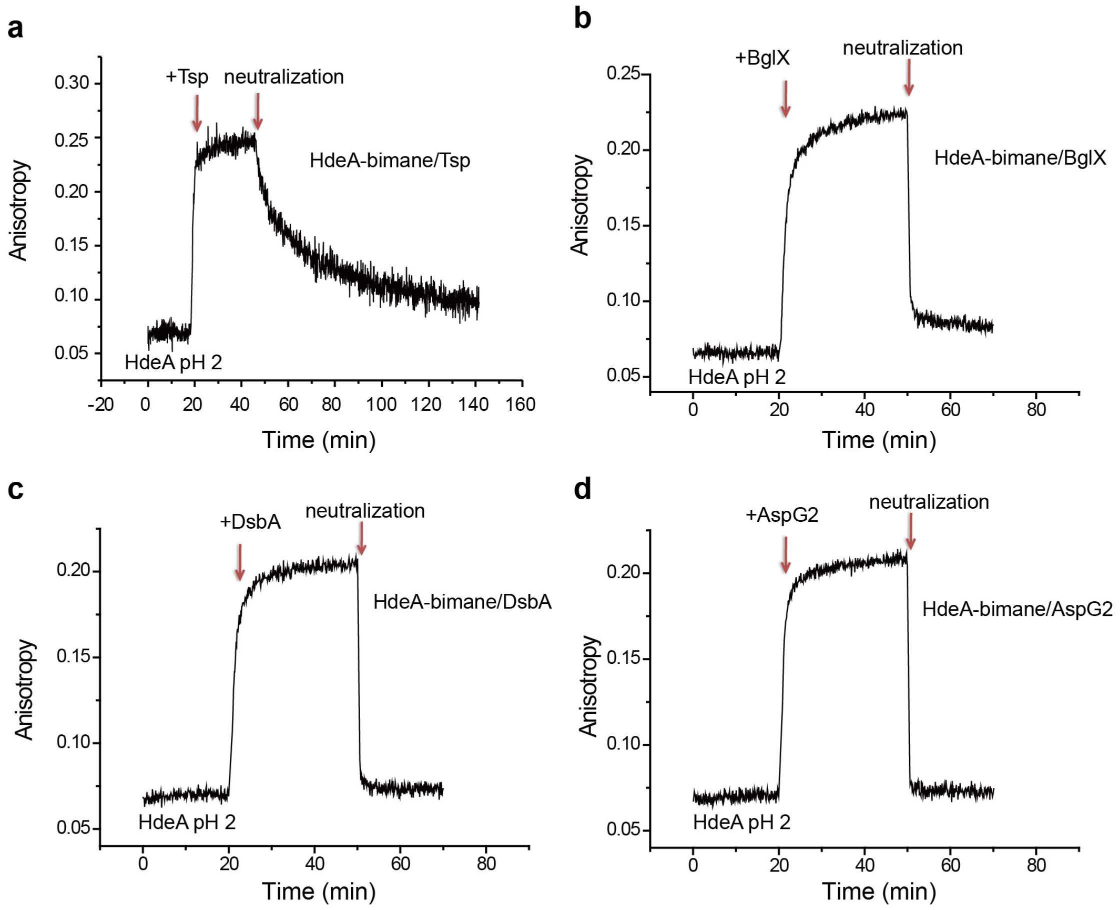

Supplementary Figure 8. Fluorescence anisotropy (FA) analysis of the interaction between HdeA and its client proteins. 3 µM HdeA (S27C)-bimane[2] was incubated alone at pH 2 for 20 min. Then 3 µM Tsp **(a)**, 1 µM BglX **(b)**, 3 µM DsbA **(c)**, or 3 µM AspG2 **(d)** was added respectively and the mixture was incubated for another 30 min. A significant increase in anisotropy was observed after the addition of the client protein, indicating the binding of HdeA to its client under acid condition. Then the solution was neutralized to pH 7 and it leads to a significant decrease in anisotropy, indicating the release of HdeA from its client after neutralization. FA of the HdeA-S27C-bimane was monitored for 140 min or 70 min during the whole process. (The representative result from 2 replicates is shown).

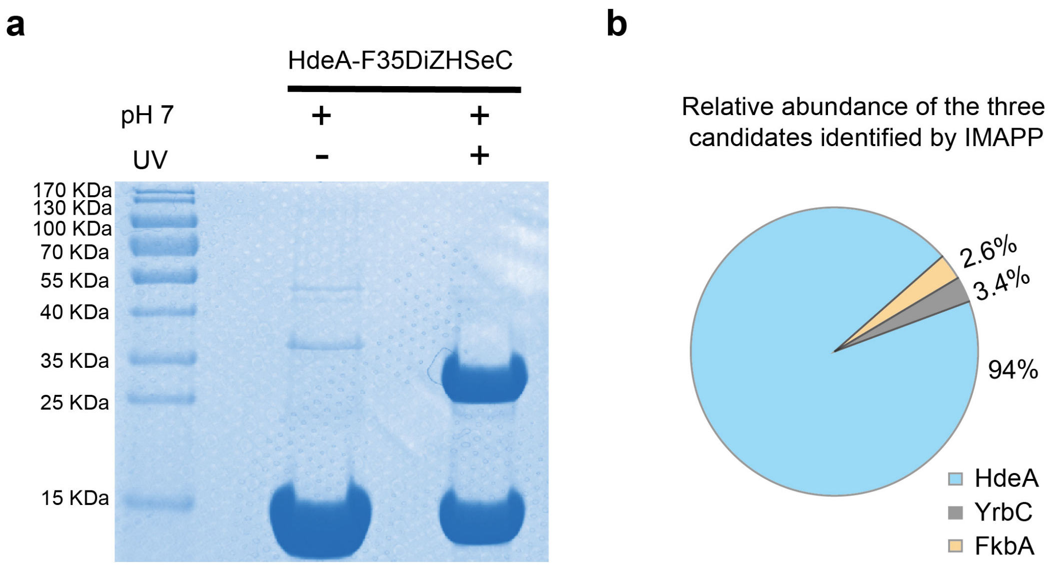

Supplementary Figure 9. Identification of HdeA interaction partners at neural pH in living cells using IMAPP strategy. (a) DiZHSeC was incorporated at residue F35 on HdeA dimer interface to photocrosslink with its interaction partner in living *E.coli* cells. The SDS-PAGE gel analysis showed that the dimer HdeA is the dominant crosslinked complex formed. When the gel bands were cut and further subjected to IMAPP analysis, only three hit proteins were identified (HdeA, FkbA, YrbC), among which HdeA was the dominant hit. **(b)** Relative abundance of the three IMAPP identified proteins. HdeA was the dominant hit among the three candidates. The relative abundance was calculated through the normalized spectral abundance factor (NSAF)[3,4]. (The representative result from 2 replicates is shown).

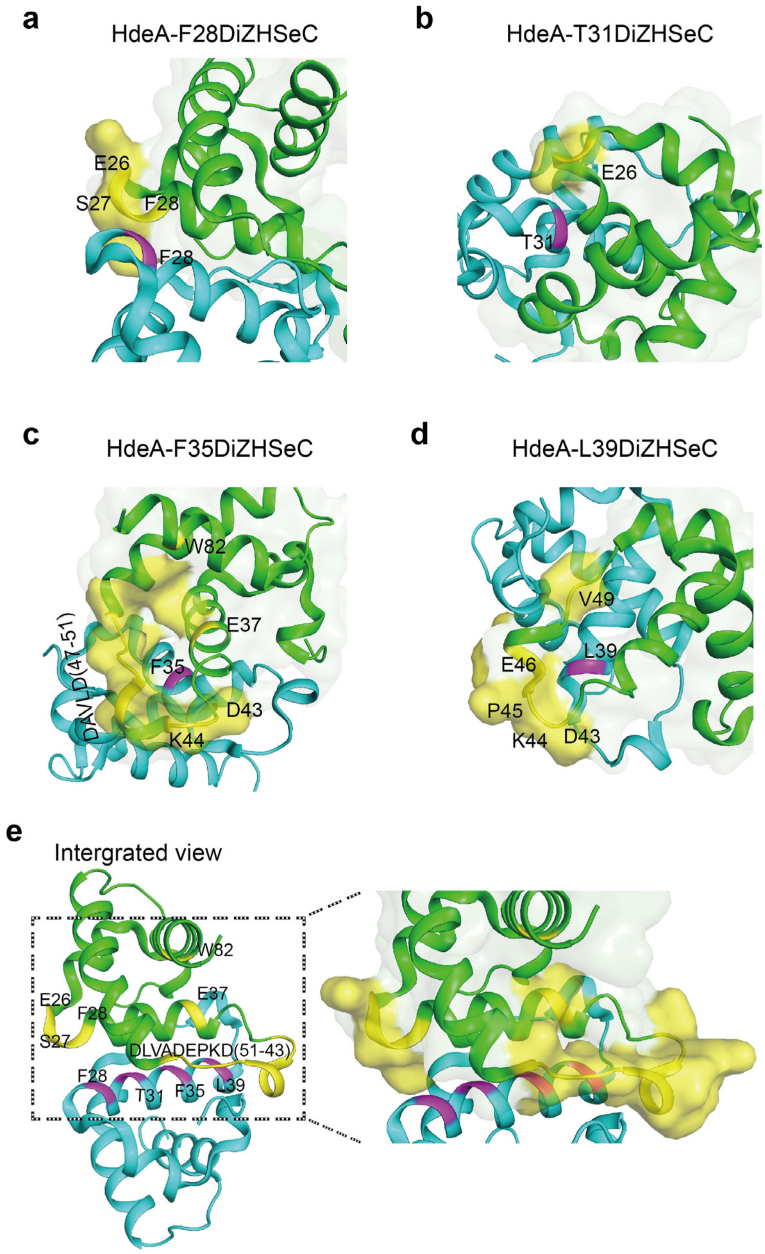

Supplementary Figure 10. Mapping of HdeA dimer interface using the IMAPP strategy. DiZHSeC was incorporated at different sites (F28, T31, F35 and L39) on HdeA to photocrosslink with WT-HdeA at pH 7. The crosslinking sites were obtained through IMAPP strategy and displayed on the crystal structure of HdeA (at pH 7) (PDB: 1DJ8)[5]. **(a)** Crosslinking sites for HdeA-F28DiZHSeC are ESF (26-28). **(b)** Crosslinking site for HdeA-T31DiZHSeC is E26. **(c)** Crosslinking sites for HdeA-F35DiZHSeC are E37, D43, K44, DAVLD (47-51) and W82. **(d)** Crosslinking sites for HdeA-L39DiZHSeC are DKPE (43-46) and V49. **(e)** Integrated view of all the incorporation sites and crosslinking sites. Close-up view of the crosslinking interface is shown in the left. Incorporation sites of DiZHSeC on the bait HdeA (colored in cyan) are colored in magentas. Crosslinking HdeA monomer is shown as a surface representation with crosslinking residues colored in yellow and the other residues colored in green. (The representative result from 3 replicates is shown).

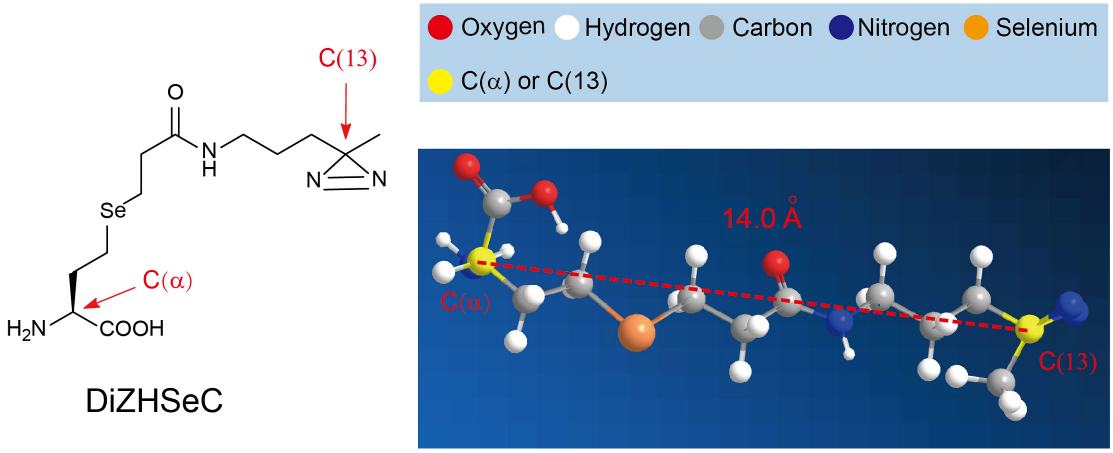

Supplementary Figure 11. Crosslinking radius of DiZHSeC as measured from a 3D structural model. The crosslinking radius was defined as the distance from the C(α) atom to the C(13) atom (the C atom on diazirine) , which was measured as 14 Å based on a structure model generated by the ChemBio Ultra 13.0 (CambrigeSoft).

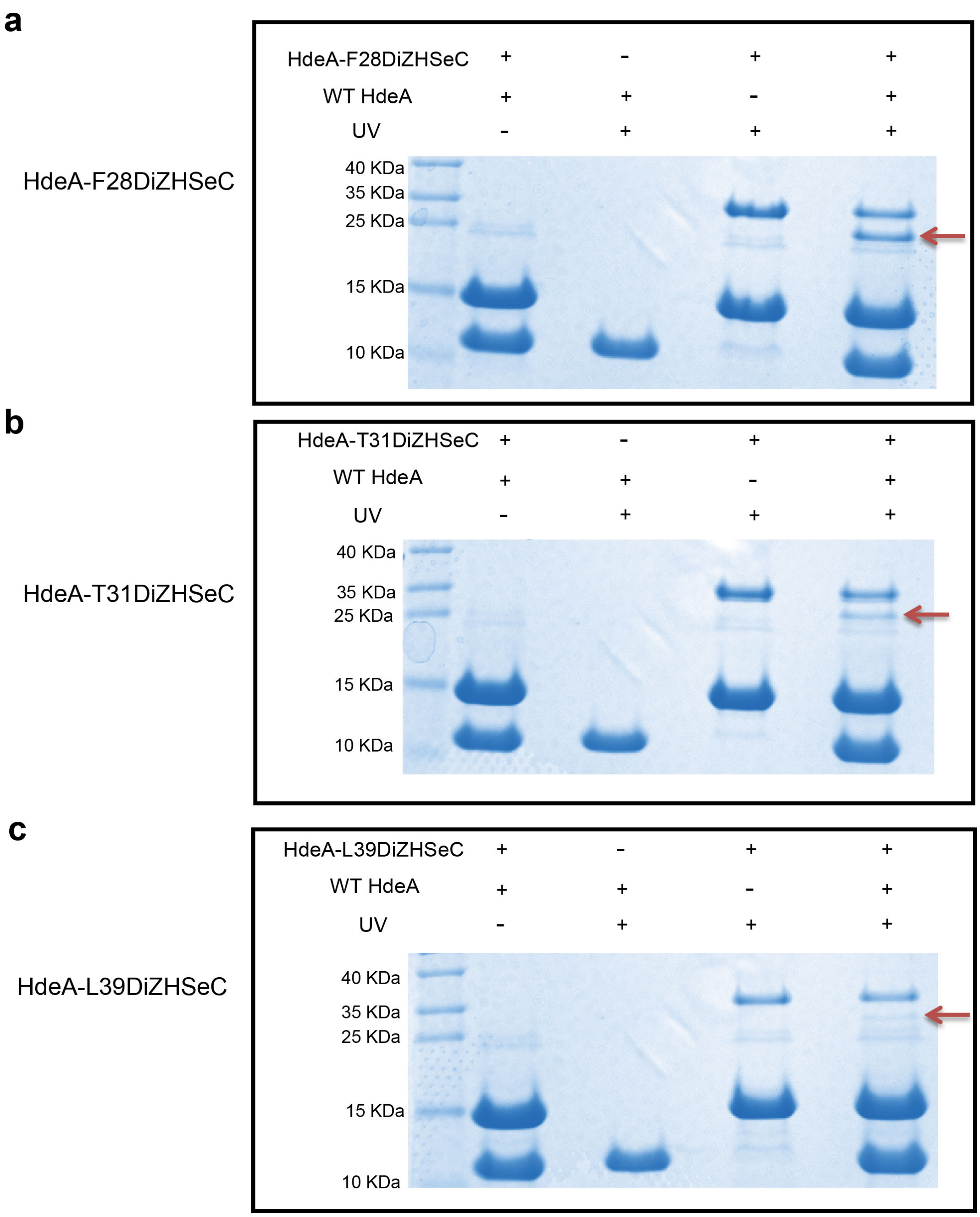

Supplementary Figure 12. Photocrosslinking of HdeA-DiZHSeC/WT-HdeA heterodimer with DiZHSeC incorporated in different sites on HdeA. DiZHSeC was incorporated at F28 **(a)**, T31 **(b)**, and L39 **(c)** position of HdeA (carrying a C terminal His-tag), respectively. These DiZHSeC-inserted HdeA variants were used to photocrosslink with WT-HdeA (carrying no tag) to create the heterodimer HdeA-F28DiZHSeC/WT-HdeA **(a)**, HdeA-T31DiZHSeC/WT-HdeA **(b)**, and HdeA-L39DiZHSeC/WT-HdeA **(c)**, respectively. The heterodimers are marked with red arrows on SDS-PAGE gels. (The representative result from 3 replicates is shown).

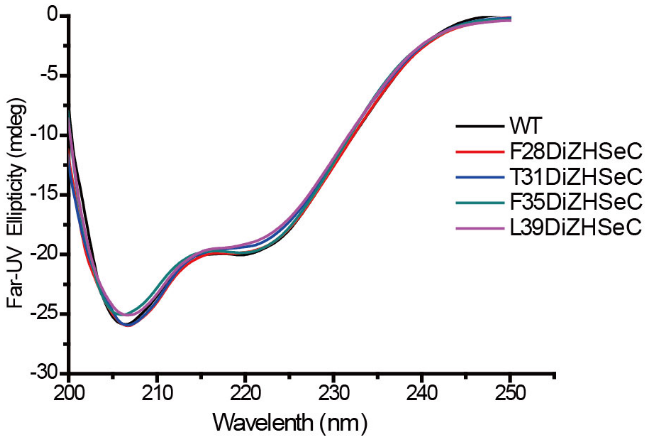

Supplementary Figure 13. Circular dichroism (CD) Analysis of WT-HdeA and HdeA-DiZHSeC variants. Far-UV CD spectra of wild-type HdeA or HdeA mutants (0.2 mg ml^{-1}) were collected in 10 mM phosphate buffer at pH 7 in a 0.1-cm path length quartz cuvette at r.t., and similar CD spectra suggests that these HdeA-DiZHSeC mutants have similar structures as that of WT-HdeA. (The representative result from 3 replicates is shown).

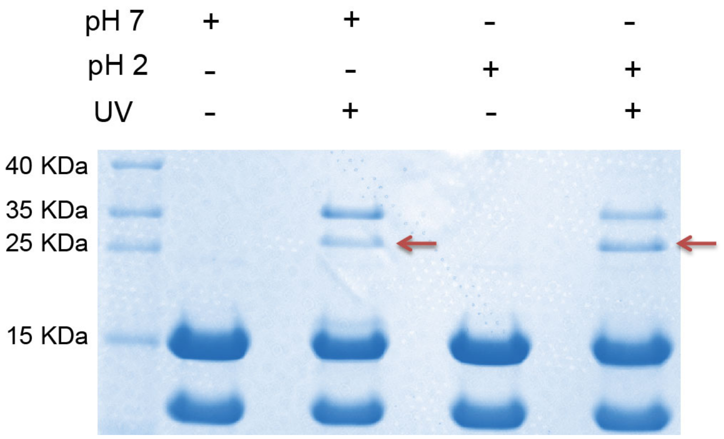

Supplementary Figure 14. Photocrosslinking of HdeA-F35DiZHSeC/WT-HdeA heterodimer at different pH. HdeA-F35DiZHSeC (carrying a C terminal His-tag) was used to photocrosslink with WT-HdeA (carrying no tag) at either pH 7 or pH 2. At both pH conditions, HdeA-F35DiZHSeC was able to capture the WT-HdeA to form the crosslinked HdeA-F35DiZHSeC/WT-HdeA heterodimer. The heterodimers are marked with red arrows on the SDS-PAGE gel. (The representative result from 3 replicates is shown).

	Crosslinked peptides and sites
pH 7	11 KPVNSWTCEDFLAVDESFQPTAVGFAEALNNK 42
	43 DKPEDAVLDVQGIATVTPAIVQACTQDK 70
	78 VKGEWDK 84
pH 2	11 KPVNSWTCEDFLAVDESFQPTAVGFAEALNNK 42
	43 DKPEDAVLDVQGIATVTPAIVQACTQDK 70

Supplementary Figure 15. Crosslinking sites illustrated the dynamic change of HdeA dimer interface from pH 7 to pH 2. DiZHSeC was incorporated at position F35 of HdeA (carrying a C-terminal His-tag) to photocrosslink with the WT-HdeA (carrying no tag) at pH 7 and pH 2 respectively. The crosslinked dimer was further subjected to IMAPP strategy to identify crosslinking peptides and sites. The crosslinking peptides and sites at pH 7 and pH 2 are listed in the table respectively. The region that is predicted to harbor the crosslinking site based on the MS/MS spectra is colored in green. The crosslinking site that could be unambiguously assigned to one specific residue based on the MS/MS spectra is colored in red. (The representative result from 3 replicates is shown).

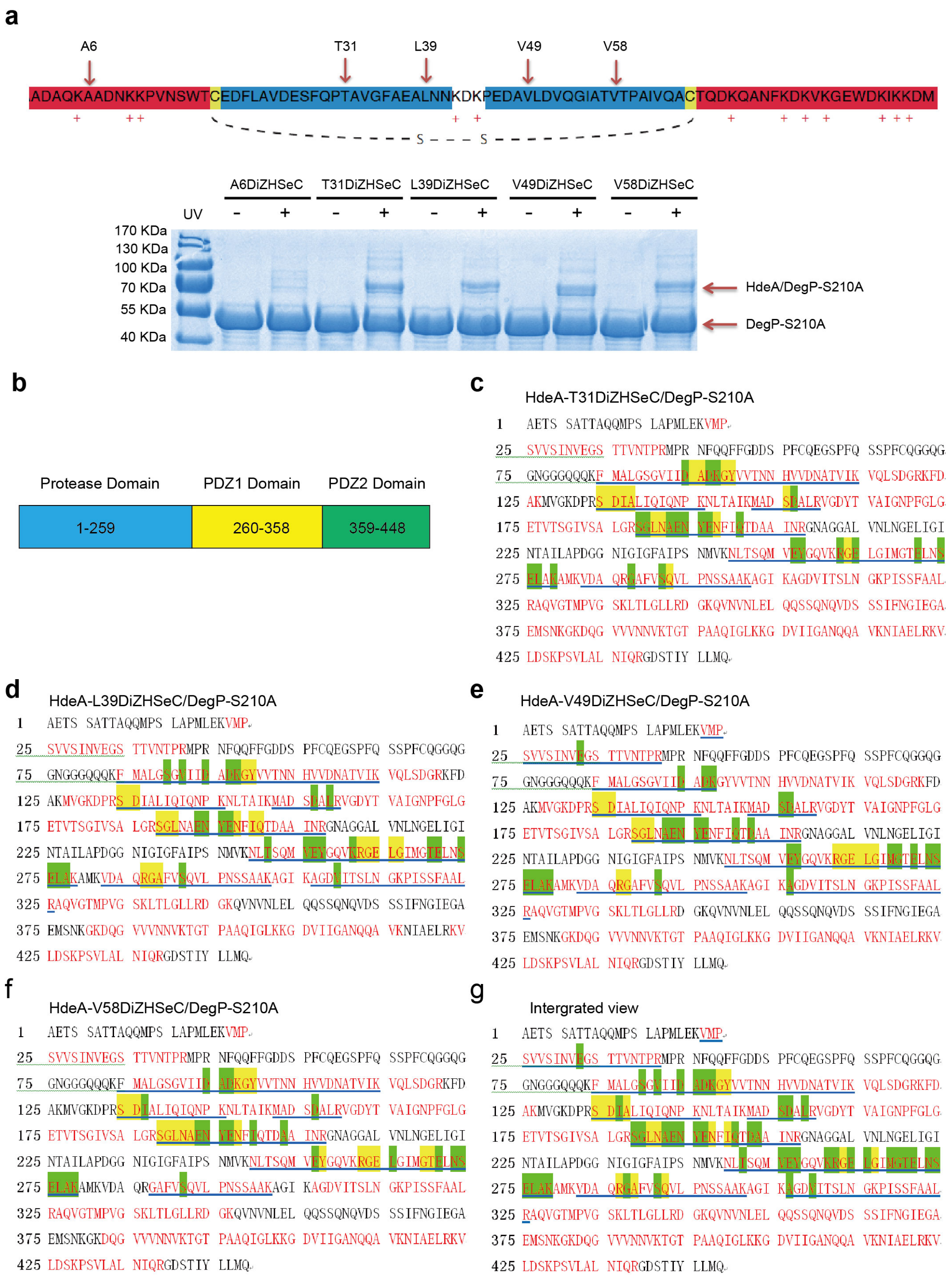

Supplementary Figure 16. Identification of a novel HdeA/DegP interaction interface. (a) HdeA interacted with DegP mainly through the two hydrophobic regions. Top: HdeA is composed of two terminal domains (colored in red) and two hydrophobic domains in the middle (colored in blue). The two hydrophobic domains are known for interactions of HdeA with its substrate proteins according to the literature[6]. Bottom: Photocrosslinking of HdeA/DegP-S210A complexes with DiZHSeC incorporated in different sites. High efficient crosslinking was observed with DiZHSeC incorporated at residue T31, L39, V49 and V58 on the two

hydrophobic regions (colored in blue) respectively, while low efficiency was observed when DiZHSeC was incorporated at residue A6 near the N-terminus (colored in red). (The representative result from 3 replicates is shown). **(b)** A sequence diagram illustrating that DegP consists of a protease domain (1-259), a PDZ1 domain (260-358) and a PDZ2 domain (359-448)[7, 8]. **(c-f)** The crosslinking peptides and sites identified by IMAPP strategy, with DiZHSeC incorporated at different sites on the two hydrophobic regions are displayed on DegP-S210A protein sequence, which are all localized on the protease domain and the PDZ1 domain. (The representative result from 2 replicates is shown). (**g**) Integrated view of all identified crosslinking peptides and sites. The crosslinking peptides are labeled in blue. All the peptides identified by LC-MS/MS analysis that could be assigned to DegP, including the crosslinking peptide and the non-crosslinking peptide, are colored in red. The region that is assigned to harbor a crosslinking site based on the MS/MS spectra is colored in yellow. The crosslinking site that could be unambiguously mapped to one specific residue based on the MS/MS spectra is colored in green.

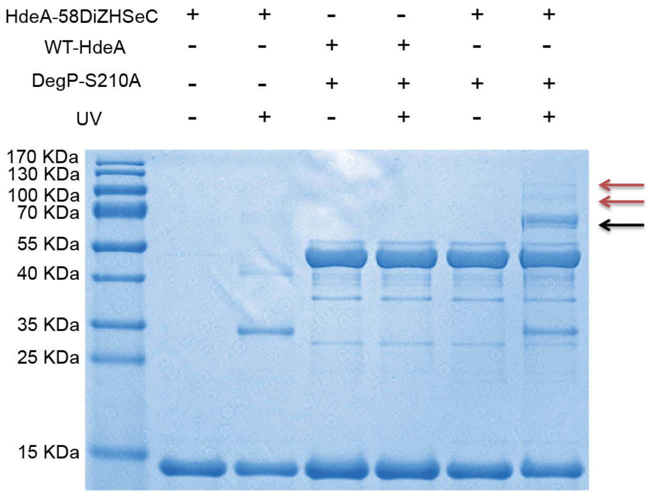

Supplementary Figure 17. Multiple HdeA may bind to a single DegP molecular under acidic condition. A solution of 50 μM HdeA-V58DiZHSeC or WT-HdeA in the present or absence of 15 μM DegP-S210A was incubated at pH 2.0 for 30 min at 37 °C. The solution was then treated with or without UV irradiation followed by the SDS-PAGE gel separation and analyzed by coomassie blue staining. Protein bands corresponding to the crosslinking complex with a 1:1 (HdeA/DegP) binding stoichiometry were marked with a black arrow. Protein bands corresponding to the crosslinking complexes with a higher (HdeA/DegP) binding stoichiometry were marked with red arrows. The immunoblotting analysis of the same gel is shown in **Fig. 5c**. (The representative result from 3 replicates is shown).

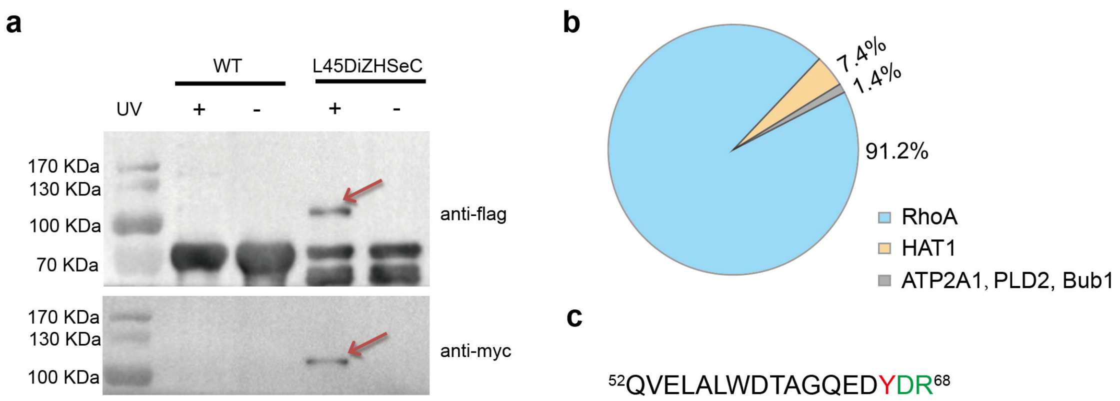

Supplementary Figure 18. Identification of specific RhoA/RTKN interaction through IMAPP. (a) Photo-capturing of the RhoA^{G14V}/RTKN-DiZHSeC complex in living cells. HEK 293T cells expressing WT-RTKN or DiZHSeC-bearing RTKN variant (containing a flag tag on its C-terminal) with the constitutively active RhoA^{G14V} (containing a myc tag on its C-terminal) were treated with and without UV irradiation for 15 min before being analyzed by immunoblotting[9,10]. The crosslinked RTKN/RhoA^{G14V} complexes were indicated by red arrows. **(b)** Relative abundance of the IMAPP identified proteins from RTKN-L45DiZHSeC photocrosslinking. The crosslinked complexes of RTKN-L45DiZHSeC were subjected to IMAPP analysis, with RhoA being the dominant hit. The relative abundance was calculated through the normalized spectral abundance factor (NSAF)[4,5]. **(c)** The crosslinking peptide and sites on RhoA. The region that is assigned to harbor the crosslinking site based on the MS/MS spectra is colored in green. The crosslinking site that could be unambiguously assigned to one specific residue based on the MS/MS spectra is colored in red (Y66). (The representative result from 2 replicates is shown).

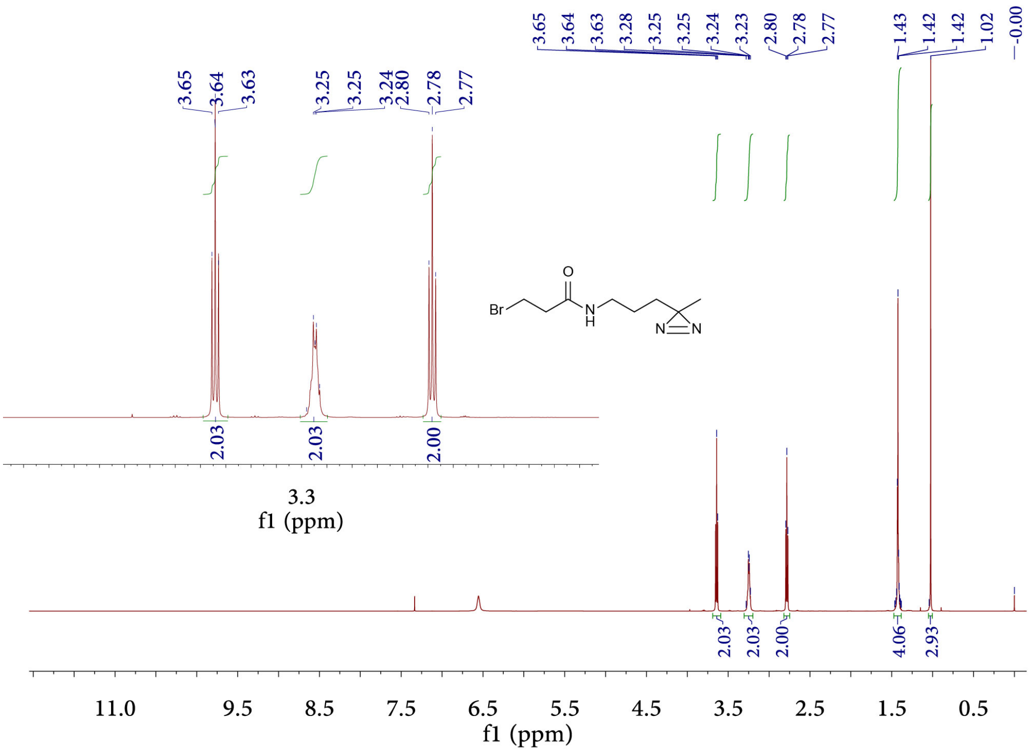

Supplementary Figure 19. ^{1}H-NMR spectrum of compound 2.

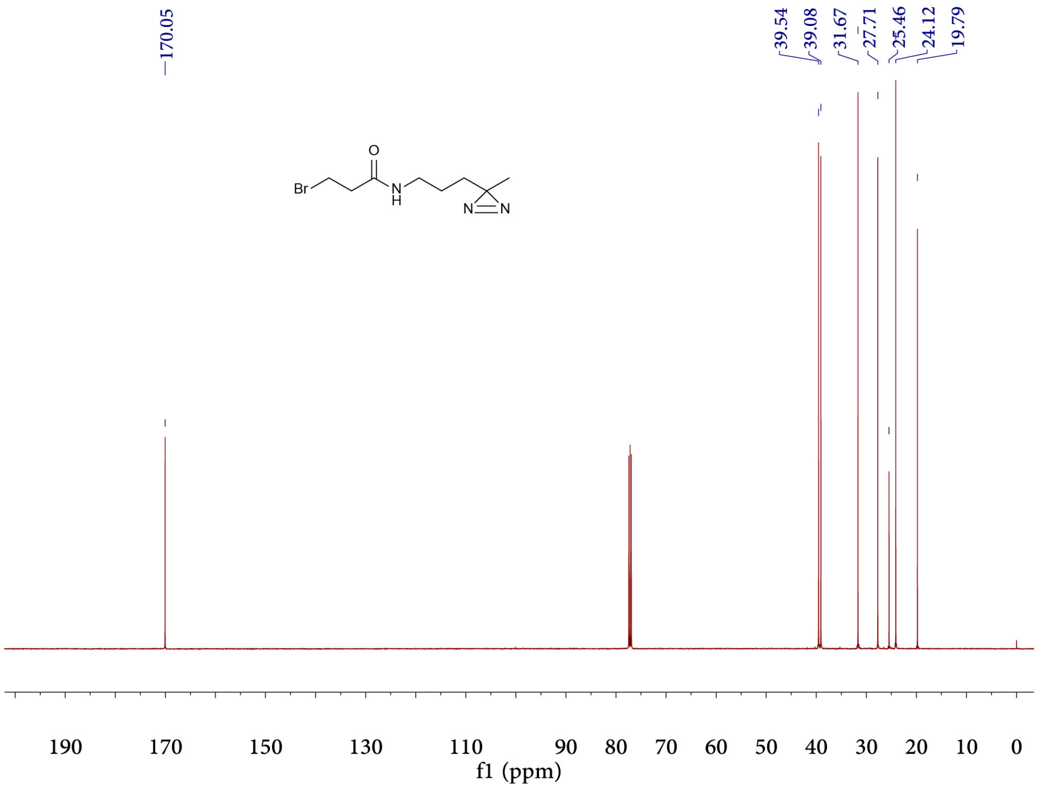

Supplementary Figure 20. ^{13}C-NMR spectrum of compound 2

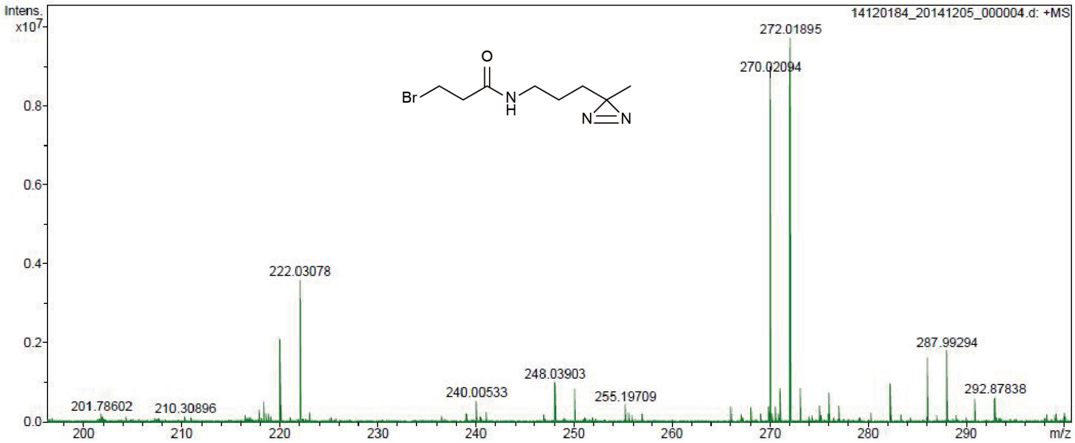

Meas. m/z	I	Formula	Score	m/z	err [mDa]	err [ppm]	mSigma	rdb	e⁻ Conf	N-Rule
248.03903	1031824	C 8 H 15 Br N 3 O	100.00	248.03930	0.3	1.1	66.4	2.5	even	ok
270.02094	8837109	C 8 H 14 Br N 3 Na O	75.32	270.02125	0.3	1.1	48.7	2.5	even	ok

Supplementary Figure 21. HRMS spectrum of compound 2

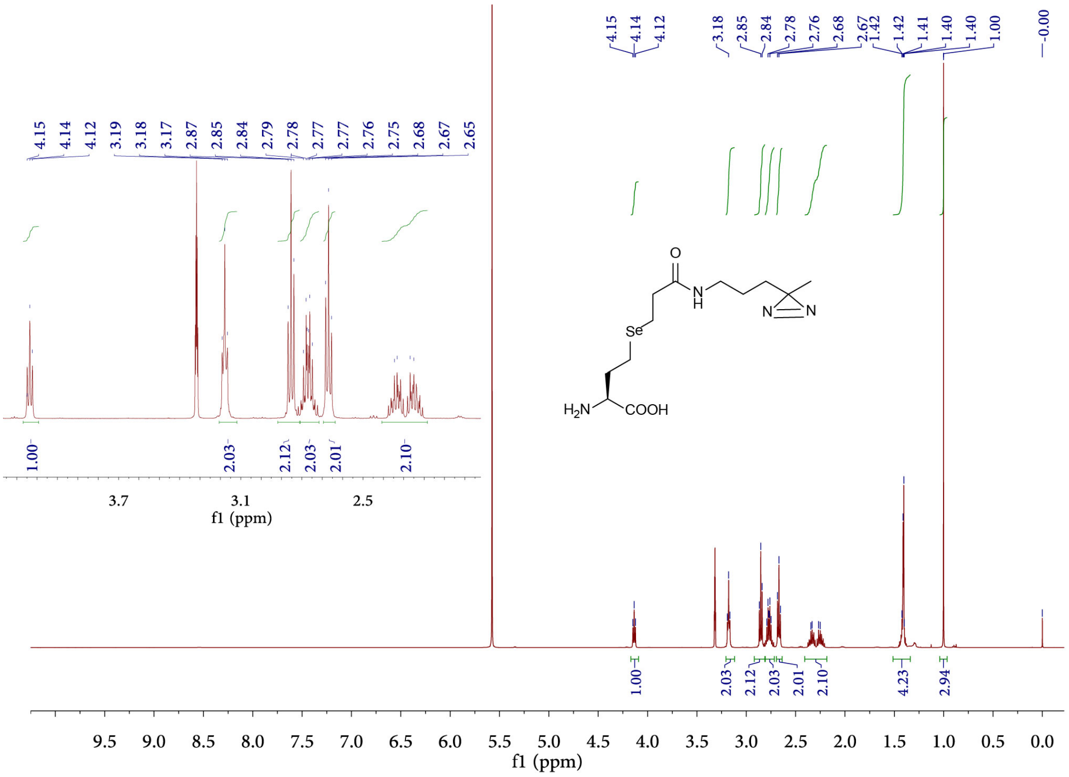

Supplementary Figure 22. ^{1}H-NMR spectrum of DiZHSeC.

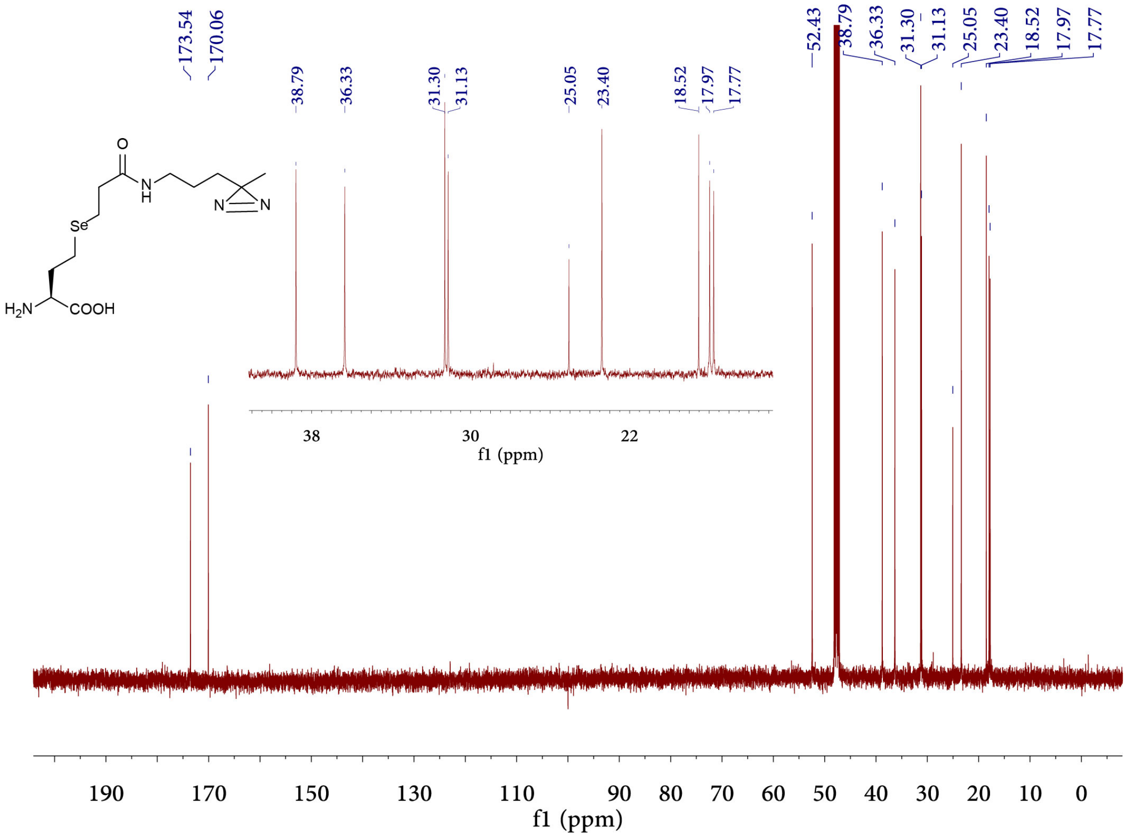

Supplementary Figure 23. ^{13}C-NMR spectrum of DiZHSeC.

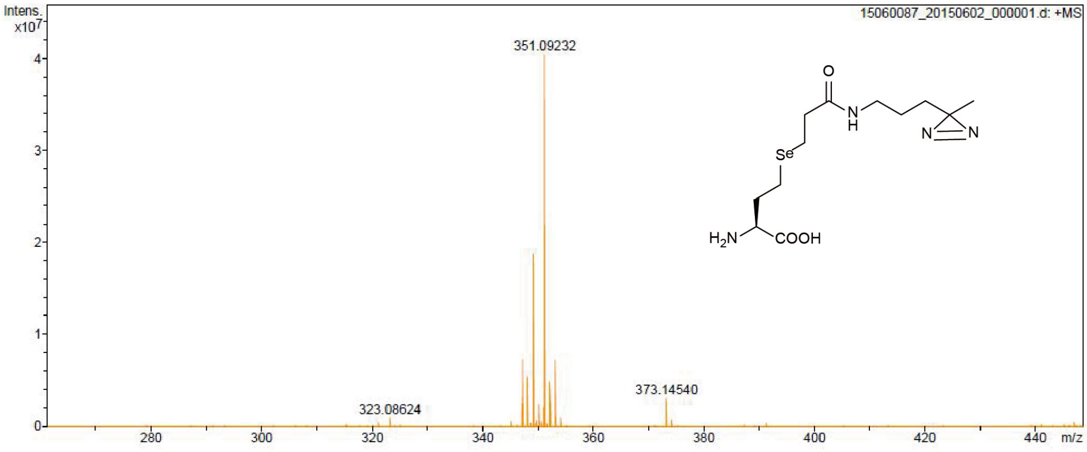

Meas. m/z	I	Formula	Score	m/z	err [mDa]	err [ppm]	mSigma	rdb	e⁻ Conf	N-Rule
351.09232	40452128	C 12 H 23 N 4 O 3 Se	100.00	351.09302	0.7	2.0	20.4	3.5	even	ok

Supplementary Figure 24. HRMS spectrum of DiZHSeC

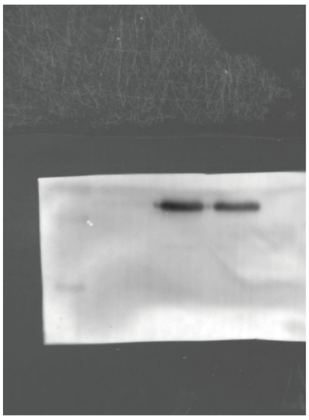

Figure 2a

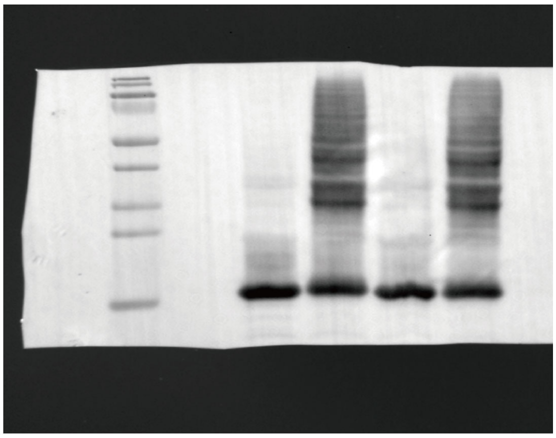

Figure 2c

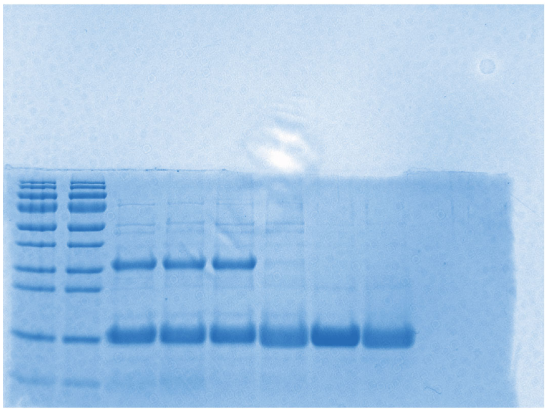

Figure 3a CBB

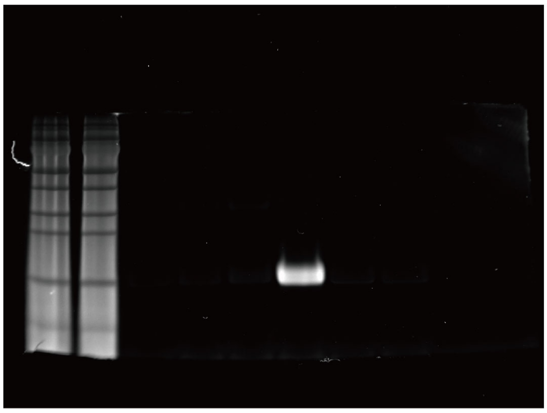

Figure 3a FL

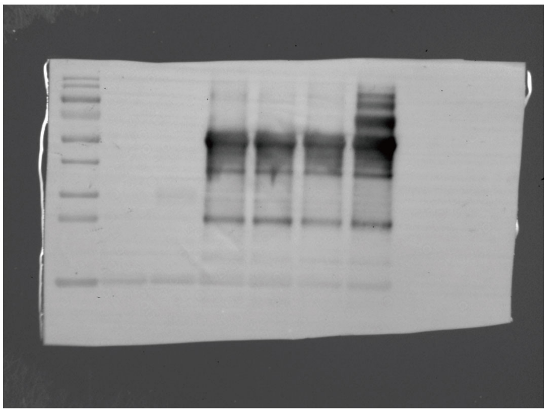

Figure 5c

Supplementary Figure 25. Full images of the blots and gels that have been presented in the main text.

Supplementary Tables

Protein name	Function	NASF Value		Ratio	Location
		Native abundance	Crosslinking abundance	Crosslinking/ Native	
AraF	L-arabinose-binding periplasmic protein	0.044647	0.121001	2.710167	Periplasm
RbsB	D-ribose-binding periplasmic protein	0.030312	0.080165	2.644672	Periplasm
HdeA	Chaperone-like protein hdeA	0.016912	0.069870	4.131408	Periplasm
DgaL	D-galactose-binding periplasmic protein	0.012841	0.045503	3.543569	Periplasm
GltI	Glutamate/aspartate periplasmic-binding protein	0.012128	0.040151	3.310611	Periplasm
GlnH	Glutamine-binding periplasmic protein	0.010002	0.012938	1.293536	Periplasm
ArgT	Lysine-arginine-ornithine-binding periplasmic protein	0.007602	0.018224	2.397293	Periplasm
FliY	Cystine-binding periplasmic protein	0.007504	0.017533	2.336437	Periplasm
TpX	Thiol peroxidase	0.007036	0.002665	0.378761	Periplasm

YtfQ	ABC transporter periplasmic-binding protein	0.006886	0.012554	1.823090	Periplasm
MalE	Maltose-binding periplasmic protein	0.006411	0.013285	2.072184	Periplasm
FkbA	FKBP-type peptidyl-prolyl cis-trans isomerase	0.006029	0.012575	2.085696	Periplasm
DppA	Periplasmic dipeptide transport protein	0.006013	0.0152023	2.5282381	Periplasm
PotD	Spermidine/putrescine-binding periplasmic protein	0.004232	0.009649	2.280007	Periplasm
SurA	Peptidyl-prolyl isomerase	0.003984	0.009153	2.297443	Periplasm
ArtI	Putative ABC transporter arginine-binding protein 2	0.003908	0.010133	2.592995	Periplasm
Agp	Glucose-1-phosphatase	0.003801	0.000181	0.047534	Periplasm
YrbC	Phospholipid ABC transporter	0.003766	0.005835	1.549422	Periplasm
LolA	Outer-membrane lipoprotein carrier protein	0.003532	0.005146	1.457001	Outer membrane
YphF	ABC transporter periplasmic-binding protein	0.003437	0.010611	3.087267	Periplasm
CH10	10 kDa chaperonin	0.003396	0.000769	0.226504	Cytosol
YdcS	Putative ABC transporter periplasmic-binding	0.003306	0.008617	2.606594	Periplasm

	protein				
MdoA	Molybdate-binding periplasmic protein	0.003167	ND	0	Periplasm
HisJ	Histidine-binding periplasmic protein	0.00313	0.006314	2.0172213	Periplasm
PstS	Phosphate-binding protein	0.00308	ND	0	Periplasm
ArtJ	ABC transporter arginine-binding protein 1	0.00303	0.003531	1.1654603	Periplasm
CysP	Thiosulfate-binding protein	0.002867	0.007726	2.695083	Periplasm
AspG2	L-asparaginase 2	0.002784	0.011793	4.236066	Periplasm
AphA	Class B acid phosphatase	0.00278	0.001732	0.622900	Periplasm
XylF	D-xylose-binding periplasmic protein	0.002701	0.003957	1.465035	Periplasm
OmpA	Outer membrane protein A	0.002632	ND	0	Outer membrane
GlpQ	Glycerophosphoryl diester phosphodiesterase	0.002598	0.010109	3.891062	Periplasm
OsmY	Osmotically-inducible protein Y	0.002507	0.0011137	0.444242	Periplasm
OppA	Periplasmic oligopeptide-binding	0.002462	0.005634	2.288466	Periplasm

	protein				
TesA	Acyl-CoA thioesterase I	0.002422	0.002332	0.962774	Periplasm
GapA	Glyceraldehyde-3-phosphate dehydrogenase A	0.002283	0.000902	0.394980	cytosolic
ZnuA	High-affinity zinc uptake system protein znuA	0.002225	0.002648	1.190007	Periplasm
UgpB	sn-glycerol-3-phosphate-binding periplasmic protein	0.002212	0.004515	2.040963	Periplasm
YbiS	L,D-transpeptidase	0.001963	0.004267	2.173927	Periplasm
TolB	Translocation protein TolB	0.001848	0.005380	2.910984	Periplasm
YtfJ	Uncharacterized protein ytfJ	0.00179	0.003447	1.925735	Periplasm
FkbB	FKBP-type 22 kDa peptidyl-prolyl cis-trans isomerase	0.001787	ND	0	Periplasm
YhjJ	Predicted zinc-dependent peptidase	0.001751	0.0043453	2.481596	Periplasm
OmpC	Outer membrane protein C	0.001742	ND	0	Outer membrane
YggE	Uncharacterized protein yggE	0.001733	0.001213	0.700124	Periplasm
NuoE	NADH-quinone oxidoreductase subunit E	0.001634	0.000899	0.550197	Inner membrane

UshA	5'-nucleotidase and UDP-sugar hydrolase	0.00155	0.005495	3.544942	Periplasm
BglX	Periplasmic beta-glucosidase	0.00152	0.005218	3.433186	Periplasm
DsbA	Thiol:disulfide interchange protein	0.001491	0.00287	1.924853	Periplasm
YdgH	Predicted protein YdgH	0.001481	0.006654	4.492850	No information
Ecotin	Serine protease inhibitor	0.001435	0.008752	6.098671	Periplasm
PotF	Putrescine-binding periplasmic protein	0.001414	0.004941	3.494327	Periplasm
TreA	Periplasmic trehalase	0.001338	0.002906	2.171534	Periplasm
PpiA	Peptidyl-prolyl cis-trans isomerase A	0.001326	0.003927	2.961771	Periplasm
OsmF	Putative osmoprotectant uptake system substrate-binding protein	0.001271	0.001590	1.251171	Periplasm
CpdB	2~,3~-cyclic-nucleotide 2~-phosphodiesterase/3~-nucleotidase	0.001198	0.004037	3.369423	Periplasm
AtpE	ATP synthase epsilon chain	0.001115	ND	0	Inner membrane
RibB	3,4-dihydroxy-2-butanone 4-phosphate synthase	0.001072	ND	0	membrane
LptA	Lipopolysaccharide	0.000943	0.0014117	1.497039	Periplasm

	export system protein				
GsiB	Glutathione-binding protein	0.000908	0.0021132	2.327345	Periplasm
MalM	Maltose operon periplasmic protein	0.000887	0.000854	0.962214	Periplasm
YbgF	Uncharacterized protein ybgF	0.000884	0.0011349	1.283810	Periplasm
AtpD	ATP synthase subunit delta	0.000876	ND	0	Periplasm
MppA	Periplasmic murein peptide-binding protein	0.000866	0.003752	4.332316	Periplasm
OsmE	Osmotically-inducible lipoprotein E	0.000865	ND	0	Periplasm
Skp	Chaperone protein skp	0.000843	ND	0	Periplasm
YpfJ	periplasmic protein that coordinates peptidoglycan synthesis and outer membrane constriction during cell division	0.00081	ND	0	Membrane
DegP	Serine endoprotease	0.000777	0.003936	5.065114	Periplasm
OmpF	Outer membrane protein F	0.000749	ND	0	Outer membrane
YgiW	Protein ygiW	0.000745	ND	0	Periplasm
OpgD	Glucans biosynthesis	0.000739	0.002505	3.390192	Periplasm

	protein D				
YebE	Inner membrane protein yebE	0.000708	ND	0	Inner membrane
YajG	Predicted lipoprotein	0.000707	ND	0	Inner membrane
Tsp	Tail-specific protease	0.000682	0.004541	6.657759	Periplasm
DegQ	Serine endoprotease	0.000681	0.003116	4.575554	Periplasm
Pal	Peptidoglycan-associated lipoprotein	0.000672	ND	0	Outer membrane
YaeT	Outer membrane protein assembly factor	0.00067	0.002027	3.024903	Outer membrane
RseB	Sigma-E factor regulatory protein rseB	0.00067	0.001995	2.976813	Periplasm
NikA	Nickel-binding periplasmic protein	0.000666	0.002207	3.314170	Periplasm
YncE	Uncharacterized protein	0.000659	0.001797	2.726512	Periplasm
OpgG	Glucans biosynthesis protein G	0.000645	0.004089	6.339076	Periplasm
ErfK	L,D-transpeptidase ErfK	0.000625	0.002527	4.043860	Periplasm
PtrA	Protease 3	0.000604	0.002443	4.045266	Periplasm
DsbC	Thiol:disulfide interchange protein	0.000575	0.000791	1.374702	Periplasm
OmpX	Outer membrane protein X	0.000567	ND	0	Outer membrane

PspE	Thiosulfate sulfurtransferase	0.000559	0.001794	3.208806	Periplasm
LivJ	Leu/Ile/Val-binding protein	0.000528	0.002135	4.043318	Periplasm
YceI	Predicted protein	0.000507	ND	0	Periplasm
OmpW	Outer membrane protein W	0.000457	ND	0	Outer membrane
AtpA	ATP synthase subunit alpha	0.000453	ND	0	Inner membrane
LivK	Leucine-specific-binding protein	0.00042	0.001921	4.574003	Periplasm
NlpC	Outer Membrane Protein Assembly Complex	0.000394	ND	0	Outer membrane
CyoA	Ubiquinol oxidase subunit 2	0.000369	ND	0	Inner membrane
YcdO	Conserved periplasmic protein	0.000362	ND	0	Periplasm
NrfA	Periplasmic cytochrome c nitrite reductase	0.000324	0.000390	1.204524	Periplasm
YcfS	L,D-transpeptidase	0.000303	0.000699	2.308752	Periplasm
SapA	Peptide transport periplasmic protein sapA	0.000283	0.000545	1.928123	Periplasm
MepA	Penicillin-insensitive murein endopeptidase	0.000283	ND	0	Periplasm

FtsY	Cell division protein	0.000273	ND	0	Inner membrane
Slt	Soluble lytic murein transglycosylase	0.00027	0.001272	4.713220	Periplasm
FepB	Ferrienterobactin-binding periplasmic protein	0.000244	ND	0	Periplasm
YfgC	Beta-barrel assembly-enhancing protease	0.000239	0.001685	7.052033	Periplasm
DsbG	Thiol:disulfide interchange protein	0.000234	0.000601	2.571644	Periplasm
NanM	N-acetylneuraminate epimerase	0.000211	0.001115	5.285437	Periplasm
CysQ	3~(2~),5~-bisphosphate nucleotidase	0.000211	ND	0	Inner membrane
AtpB	ATP synthase subunit beta	0.000211	ND	0	Inner membrane
OmpN	Outer membrane protein N	0.000206	ND	0	Outer membrane
PpiD	Peptidyl-prolyl cis-trans isomerase D	0.000187	ND	0	Inner membrane
YgiS	Putative binding protein ygiS	0.000181	0.000836	4.623463	Periplasm
YnjE	Putative thiosulfate sulfurtransferase ynjE	0.000178	0.000514	2.891082	Periplasm

YfgL	Lipoprotein	0.000148	ND	0	Outer membrane
BtuF	Vitamin B12-binding protein	0.000146	0.000701	4.803458	Periplasm
MetQ	D-methionine-binding lipoprotein MetQ	0.000143	0.000688	4.813747	Membrane
YcbB	L,D-transpeptidase	0.0000630	0.000364	5.775855	Membrane
YfhM	α-macroglobulin	2.344E-05	0.000316	13.47702	Inner membrane
FdoG	Formate dehydrogenase-O major subunit	ND	0.001064	/	Periplasm
AmpC	Beta-lactamase	ND	0.000395	/	Periplasm
AmiC	N-acetylmuramoyl-L-alanine amidase AmiC	ND	8.947E-09	/	Periplasm
CreA	conserved protein CreA	ND	0.002376	/	Cytosol

Supplementary Table 1. The "native" and "crosslinking" abundance of all the crosslinked proteins as well as the non-crosslinked envelope proteins from *E. coli* periplasmic extraction. The *E. coli* periplasmic extraction was isolated and analyzed by LC-MS/MS analysis according to the Supplementary Methods. The relative protein abundance was determined using the NASF method with all the proteins identified either in the native periplasmic extraction or in the crosslinking group[3,4]. All the identified HdeA-crosslinked proteins from this study are listed, which include 50 envelope proteins (colored in red) and 2 cytosolic proteins (resulted from non-specific crosslinking as discussed in the maintext, colored in blue). Because HdeA is a periplasmic chaperone that is expected to only interact with the envelope proteins (located in periplasm or outer and inner membrane), the abundance of all non-crosslinked envelop proteins from the native *E. coli* periplasmic extraction are also listed for comparison (colored in black). The non-crosslinked proteins from cytosol or with unknown location are not included in the table. All the proteins are ranked according to their native abundance (NSFA values, from high to low). ND: not

detected. The table shows that the HdeA clients identified by IMAPP spans the whole range of protein abundance in cell envelope. Majority of our identified HdeA client proteins (colored in red) were significantly enriched in the "crosslinking group", whereas most of the proteins colored in black were not enriched. Some highly abundant proteins such as Tpx and Agp were not crosslinked and had a very low abundance in the crosslinking group. Meanwhile, some other identified client proteins with extremely low abundance such as FdoG were efficiently enriched and showed moderate abundance in the crosslinking group. Taken together, these data and analysis indicate that our photocrosslinking results reflect the intrinsic nature and binding preference of HdeA rather than the non-specific interactions based only on native protein abundance. (The representative result from 2 replicates is shown).

Protein	Function	Subcellular location
FdoG	Formate dehydrogenase-O major subunit	Periplasm
AraF	L-arabinose-binding periplasmic protein	Periplasm
UshA	5'-nucleotidase and UDP-sugar hydrolase	Periplasm
SurA	Peptidyl-prolyl isomerase SurA	Periplasm
OppA	Periplasmic oligopeptide-binding protein	Periplasm
MalE	Maltose-binding periplasmic protein	Periplasm
DppA	Periplasmic dipeptide transport protein	Periplasm
OpgG	Glucans biosynthesis protein G	Periplasm
CpdB	2~,3~-cyclic-nucleotide 2~-phosphodiesterase/3~-nucleotidase	Periplasm
DegP	Serine endoprotease	Periplasm
RbsB	D-ribose-binding periplasmic protein	Periplasm
FkbA	FKBP-type peptidyl-prolyl cis-trans isomerase FkbA	Periplasm
GlpQ	Glycerophosphoryl diester phosphodiesterase	Periplasm
TolB	translocation protein TolB	Periplasm
YaeT	Outer membrane protein assembly factor YaeT	Outer membrane
MetQ	D-methionine-binding lipoprotein MetQ	Periplasm
FliY	Cystine-binding periplasmic protein	Periplasm
ArgT	Lysine-arginine-ornithine binding periplasmic protein	Periplasm
DgaL	D-galactose-binding periplasmic protein	Periplasm
DegQ	Serine endoprotease	Periplasm
PotF	Putrescine-binding periplasmic protein	Periplasm
YhjJ	Predicted zinc-dependent peptidase	Periplasm
BglX	Periplasmic beta-glucosidase	Periplasm
Tsp	Tail-specific protease	Periplasm
YfhM	Uncharacterized lipoprotein YfhM	Inner membrane
Slt	Soluble lytic murein transglycosylase	Periplasm
YtfQ	ABC transporter periplasmic-binding protein YtfQ	Periplasm
XylF	D-xylose-binding periplasmic protein	Periplasm
YdcS	Putative ABC transporter periplasmic-binding protein	Periplasm

	YdcS	
AspG2	L-asparaginase 2	Periplasm
YncE	Conserved protein YncE	Periplasm
PotD	Spermidine/putrescine-binding periplasmic protein	Periplasm
YbiS	L,D-transpeptidase	Periplasm
YrbC	Phospholipid ABC transporter	Periplasm
DsbA	Thiol:disulfide interchange protein DsbA	Periplasm
Skp	Chaperone protein Skp	Periplasm
YphF	ABC transporter periplasmic-binding protein YphF	Periplasm
MppA	Periplasmic murein peptide-binding protein	Periplasm
YdgH	Predicted protein YdgH	No information
AmiC	N-acetylmuramoyl-L-alanine amidase AmiC	Periplasm
GlnH	Glutamine-binding periplasmic protein	Periplasm
AmpC	Beta-lactamase	Periplasm
GltI	Glutamate/aspartate periplasmic-binding protein	Periplasm
PtrA	Protease 3	Periplasm
CysP	Thiosulfate-binding protein	Periplasm
TreA	Periplasmic trehalase	Periplasm
GsiB	Glutathione-binding protein GsiB	Periplasm
YfgC	Beta-barrel assembly-enhancing protease	Periplasm
Ecotin	Serine protease inhibitor	Periplasm
ArtI	Putative ABC transporter arginine-binding protein 2	Periplasm

Supplementary Table 2. List of HdeA client proteins identified by IMAPP strategy. The protein names, functions, and subcellular locations of the 50 HdeA client proteins identified by IMAPP are listed, including those 22 proteins (protein names are colored in black) that have been reported in previous studies[6, 11] and those 28 proteins (protein names are colored in blue) that are newly identified.

Protein	Function	Crosslinking peptides and sites
FdoG	Formate dehydrogenase-O major subunit	GADAPGIALTDGEILSGIFLR
AraF	L-arabinose-binding periplasmic protein	DNFKEELEK
		VIAVDDQFVNAK
		SSEMLYNWVAK
		DVEPPKFTEVTDVVLITR
		QPEEPWFQTEWK
		ESAVMAITANELDTAR
		AADIIGIGINGVDAVSELSK
		SNDIPGAFDAANSMLVQHPEVK
		DLGFEVIK
		IAVPDGEK

		ATEGQGFK
		GFVICTPDPK
		AQATGFYGSLLPSPDVHGYK
		DLGFEVIKIAVPDGEK
		GWDVKESAVMAITANELDTAR
UshA	5'-nucleotidase and UDP-sugar hydrolase	VLYTPEIAENQQMISLLSPFQNK
		SSPLDVSVYEPK
		NEYGEYGLAAQK
		IGNPEYFTDIEFR
SurA	Peptidyl-prolyl isomerase SurA	IQELPGIFAQALSTAK
		ISDEQLDQAIANIAK
		FSEEAASWMQEQR
		KFSEEAASWMQEQR
OppA	Periplasmic oligopeptide-binding protein	TVINQVTYLPIASEVTDVNR
		SPAFDSIMAETLK
		AEQQLDKDSAIVPVYYYVNAR
MalE	Maltose-binding periplasmic protein	EFLENYLLTDEGLEAVNK
		IAATMENAQK
		FGGYAQSGLLAEITPDK
		AGLTFLVDLIK
		VNYGVTVLPTFK
		GEIMPNIPQMSAFWYAVR
		GETAMTINGPWAWSNIDTSK
		TWEEIPALDKELK
DppA	Periplasmic dipeptide transport protein	HHFENVSIE
		MAEMIQADWAK
		ELNADDVVFSFDR
		NECQVMPYPNPADIAR
		IGTTEVIPGLAEKWEVSEDGK
		SINLMEMPGLNVGYLSYNVQK
		VSGGSYEYFEGMGLPELISEVK
		ELNADDVVFSFDR
OpgG	Glucans biosynthesis protein G (periplasm)	GLAIDTALPSGEEFPR
		DKNDEIVSMLGASYFR
		LEFYHQGMYFDTPVK
		KLPEDTPVTAQTSIGDNGEIVESTVR
		INEVTATAVK
CpdB	2~,3~-cyclic-nucleotide 2~-phosphodiesterase/3~-nucleotidase	SADNMYSYLALVQDDPTVQVVNNAQK
DegP	Serine endoprotease	SDIALIQIQNPK
		SGLNAENYENFIQTDAAINR
RbsB	D-ribose-binding periplasmic	FNVLASQPADFDR

	protein	MANQANIPVITLDR
		SDVMVVGFDGTPDGEK
		GLNVMQNLLTAHPDVQAVFAQNDEMALGALR
		ELANVQDLTVR
		VIELQGIAGTSAAR
		GEGFQQAVAAHK
		ILLINPTDSDAVGNAVK
		EADKLGYNLVVLDSQNNPAK
		LGYNLVVLDSQNNPAK
FkbA	FKBP-type peptidyl-prolyl cis-trans isomerase FkbA	LSDQEIEQTLQAFEAR
		TSSTGLVYQVVEAGK
		LDGVIPGWTEGLK
GlpQ	Glycerophosphoryl diester phosphodiesterase	VHTFEEEIEFVQGLNHSTGK
		SDKLPEYTPDVNQLYDALYNK
TolB	translocation protein TolB	LAYVTFESGR
YaeT	Outer membrane protein assembly factor YaeT	ALFATGNFEDVR
		LAGDLETLR
MetQ	D-methionine-binding lipoprotein MetQ	LKDGVGLLPTVLDVVENPK
		IVELEAPQLPR
		DGIFVEDKESPYVNLIVTR
		VGVIVGAEQQVAEVAQK
		FVQAYQSDEVYEAANK
FliY	Cystine-binding periplasmic protein	VGVGLGTNYEEWLR
		LAALDLVK
		IDAILVDR
ArgT	Lysine-arginine-ornithine binding periplasmic protein	LDAALQDEVAASEGFLK
		QQEIAFSDK
		HVGVLQGSTQEAYANETWR
DgaL	D-galactose-binding periplasmic protein	ALAINLVDPAAAGTVIEK
		SSIPVFGVDALPEALALVK
		TEQLQLDTAMWDTAQAK
		ALDSYDK
		ESGIIQGDLIAK
		QNDQIDVLLAK
DegQ	Serine endoprotease	TLAQQLIDFGEIK
		SGLNLEGLENFIQTDASINR
PotF	Putrescine-binding periplasmic protein	VVYDVFDSNEVLEGK
YhjJ	Predicted zinc-dependent peptidase	LLVNTGSLAESTQQSGYSHAIPR
BglX	Periplasmic beta-glucosidase	ASEGFGEDTYLTSTMGK
		AMQDQVMELSR
Tsp	Tail-specific protease	GPLVVLVDR

		LDDVVALIK
		ALVVGEPTFGK
		SGDLTAFEPELLK
		LDVFYDLYNLAQK
		LAQTNSEDVFSLAMTAFAR
		VGVLDIPGFYVGLTDDVK
YfhM	Uncharacterized lipoprotein yfhM	EAVSALPGFEFGDIAAENLSR
Slt	Soluble lytic murein transglycosylase	IDAVAFVESIPFSETR
YtfQ	ABC transporter periplasmic-binding protein YtfQ	DAEIPVFLLDR
		SLYMTTVTADNILEGK
		DILTGSIDGVPDIYK
XylF	D-xylose-binding periplasmic protein	VVGDQWVDGWLPENALK
YdcS	Putative ABC transporter periplasmic-binding protein YdcS	LDIIAWPGYIER
		AEGQPVATVFPK
AspG2	L-asparaginase 2	VGIVYNYANASDLPAK
		VGVENLVNAVPQLK
		DPQQIQQIFNQY
YncE	Conserved protein YncE	AAEVLVVDTR
		VAAPESLAVLFNPAR
PotD	Spermidine/putrescine-binding periplasmic protein	QVAETIGYPTPNLAAR
		VIYSTYESNETMYAK
YbiS	L,D-transpeptidase	SVQTVTGQPDVDQVVLDEAIK
		YIEVHNPLSTTEAQFEGQEIVPITLTK
YrbC	Phospholipid ABC transporter	QNEWGTLLR
		TIVDQELLPYVQVK
DsbA	Thiol:disulfide interchange protein DsbA	GEEYDAAWNSFVVK
		DLTQAWAVAMALGVEDK
Skp	Chaperone protein Skp	AQAFEQDR
YphF	ABC transporter periplasmic-binding protein YphF	NVDAIILSAVSENGSSR
MppA	Periplasmic murein peptide-binding protein	VLAQASTENTVK
		ASWVGDYNEPSTFLTLLTSTHSGNISR
		DIPGQVYTPPQLGTYYYAFNTQK
		GYPINNPEDVAYSR
YdgH	Predicted protein YdgH	KVEIPGVATTASPSSEVGR
AmiC	N-acetylmuramoyl-L-alanine amidase AmiC	NEDIFIPLQVR
		TATFQQEVAESILAGIK

GlnH	Glutamine-binding periplasmic protein	LVVATDTAFVPFEFK
		QFPNIDNAYMELGTNR
		AVGDSLEAQQYGIAFPK
		ADAVLHDTPNILYFIK
AmpC	Beta-lactamase	VDAAWQILNALQ
GltI	Glutamate/aspartate periplasmic-binding protein	NLNMNFELSDEMK
		AVVVTSGTTSEVLLNK
		AVAFMMDDALLAGER
		VVGYSQDYSNAIVEAVK
		KLMDDTIAQVQTSGEAEK
PtrA	Protease 3	TDELITYLIGNR
		TEEQLGYAVFAFPMSVGR
CysP	Thiosulfate-binding protein	TNILAEFPVAWVDK

Supplementary Table 3. List of the crosslinking peptides and sites on HdeA client proteins identified by IMAPP strategy. The crosslinking sites identified in two of the three replicates are listed in the table. The region that is assigned to harbor the crosslinking site based on the MS/MS spectra is colored in green. The crosslinking site that could be unambiguously mapped to a single specific residue based on the MS/MS spectra is colored in red.

Incorporation sites	Crosslinking sites	Distance Å
F28DiZHSeC	E26	12.6
	S27	9.8
	F28	9.6
T31DiZHSeC	E26	13.5
F35DiZHSeC	E37	7.8
	D43	14.6
	K44	14.2
	D47	13.1
	A48	9.7
	V49	9.8
	L50	5.5
	D51	10.5
	W82	11.7
L39DiZHSeC	D43	9.3
	K44	9.4
	P45	7.4
	E46	11.1
	V49	10.5

Supplementary Table 4. The distance from the incorporated DiZHSeC (Cα atom) to the closest C atom of the crosslinking residues measured from the crystal

structure of HdeA dimer (pH 7). The region that is predicted to harbor the crosslinking site based on the MS/MS spectra is colored in green. The crosslinking site that could be unambiguously assigned to one specific residue based on the MS/MS spectra is colored in red.

Supplementary Methods

Synthesis of DiZHSeC

The overall synthetic route of DiZHSeC is shown in Supplementary Fig. 1. Compound **1** was synthesized as described previously[6]. A mixture of compound **1** (950 mg, 8.4 mmol) and trimethylamine (1.17 ml, 8.4 mmol) was dissolved in 30 ml CH_2Cl_2 at 0 °C. 7.6 mmol 3-bromopropionyl chloride was dissolved in 5 ml CH_2Cl_2 and added to the solution mentioned above dropwise over 20 min. The temperature was then allowed to rise to r.t. gradually. After overnight stirring, the solvent was rotary vaporized to give a white solid. The solid was subsequently dissolved in 40 ml CH_2Cl_2 and washed with 1 M HCl, H_2O and brine successively and then dried over Na_2SO_4, evaporated under vacuum. The crude product was further purified through HPLC and gave compound **2** as a white solid (1.2 g, 63.2%).

To a nitrogen-protected solution of selenomethionine **3** (788 mg, 4 mmol) in liquid ammonia (~ 30 mL) cooled in a dry ice/acetone bath was added sodium metal (276 mg, 12 mmol) in small pieces over 40 min. The solution was stirred for another 1 h at -78 °C. Then, the bath temperature was gradually raised up to r.t. and excessive ammonia was blown away with a gentle stream of nitrogen inside a well-ventilated fume hood. Residual ammonia was removed under vacuum inside a well-ventilated fume hood to give the crude product **4**. Product **4** was used without further purification. The crude product was carefully dissolved with 15 ml degassed ice-cold water under nitrogen atmosphere. Compound **2** (1.02 g, 4 mmol) was dissolved with 9 ml degassed ethanol and added to the solution of **4** dropwise over 10 min under nitrogen atmosphere at 0 °C. The temperature was gradually raised up to r.t., and the mixture was allowed for stirring overnight. Then, the pH of the solution was adjusted to 5~6 to precipitate the crude product as a light yellow solid. The solid was further purified by HPLC and gave the product DiZHSeC as a white solid (600 mg, 42.8%).

Preparation and analysis of E.coli periplasmic proteome.

The protocol for isolation of *E. coli* periplasmic proteins was performed according to the Epicentre PeriPreps™ Periplasting Kit. In brief, a 10 ml volume of *E.coli* cells bearing the HdeA-V58DiZHSeC were harvested by centrifugation at 4000 rpm for 10 min and the supernatant was discarded. The pellet was re-suspended in 0.5 ml of periplasting buffer (20% sucrose, 1 mM EDTA, and 30000 unit ml^{-1} lysozyme). The

sample was incubated on ice for 5 min followed by quickly adding of 0.5 ml of ice-cold water. Then the sample was incubated on ice for additional 5 min followed by centrifugation at 12000 rpm for 2 min. The supernatant was recovered as the periplasmic fraction. Then, the proteome was separated by the SDS-PAGE gel and the corresponding protein bands were excised and cut into pieces. To obtain the crosslinking proteome, the *E. coli* cells were subjected to the protocol described above after photocrosslinking, followed by the Ni-NTA purification procedure. The crosslinking proteome were separated by the SDS-PAGE gel and the corresponding protein bands were excised and cut into pieces. The gel pieces were dehydrated in acetonitrile, incubated in buffer I (10 mM DTT, 50 mM ammonium bicarbonate) at 56 °C for 30 min, and were further incubated in buffer II (55 mM iodoacetamide, 50 mM ammonium bicarbonate) at ambient temperature for 1 h in the dark before being dehydrated. Then the samples were in-gel digested with sequencing grade trypsin (5 ng μl^{-1} trypsin, 50 mM ammonium bicarbonate, pH 8.0) overnight at 37 °C. The resulting peptides were extracted twice with 5% formic acid/50% acetonitrile in water, and then vacuum-centrifuged to dryness. The extracted peptides were reconstituted in 0.2% formic acid, loaded onto a 100 μm x 2 cm pre-column and separated on a 75 μm x 20 cm capillary column both of which were packed in-house with 4 μm C18 bulk materials (InnosepBio, China). An Easy nLC 1000 system (Thermo Scientific, USA) was used to generate the following HPLC gradient: 5-30% B in 120 min, 30-75% B in 4 min, then held at 75% B for 20 min (A = 0.1% formic acid in water, B = 0.1% formic acid in acetonitrile). The eluted peptides were sprayed into an LTQ-Orbitrap-Elite mass spectrometer (Thermo Scientific, USA) equipped with a nano-ESI source. The mass spectrometer was operated in data-dependent mode with one MS scan in FT mode at a resolution of 120000 followed by 10 CID (Collision Induced Dissociation) MS/MS scans in the ion trap for each cycle. Raw data files produced in the Xcalibur software (Thermo Scientific) were transformed to mgf files through MSConvert and then searched with Mascot V.2.3.02 (Matrix Science) against SwissProt 57.15 (515,203 sequences; 181,334,896 residues) *Escherichia coli* database (22,646 sequences).

Supplementary References

1. Yu, Z. & Lin, Q. Design of Spiro[2.3]hex-1-ene, a Genetically Encodable Double-Strained Alkene for Superfast Photoclick Chemistry. *J. Am. Chem. Soc.* **136**, 4153-4156 (2014).
2. Tapley, T.L., Franzmann, T.M., Chakraborty, S., Jakob, U. & Bardwell, J.C.A. Protein refolding by pH-triggered chaperone binding and release. *Proc. Natl. Acad. Sci. U. S. A.* **107**, 1071-1076 (2010).
3. Florens, L. et al. Analyzing chromatin remodeling complexes using shotgun proteomics and normalized spectral abundance factors. *Methods* **40**, 303-311 (2006).
4. Paoletti, A.C. et al. Quantitative proteomic analysis of distinct mammalian Mediator complexes using normalized spectral abundance factors. *Proc. Natl. Acad. Sci. U. S. A.* **103**, 18928-18933 (2006).
5. Gajiwala, K.S. & Burley, S.K. HDEA, a periplasmic protein that supports acid resistance in pathogenic enteric bacteria1. *J. Mol. Biol.* **295**, 605-612 (2000).
6. Zhang, M. et al. A genetically incorporated crosslinker reveals chaperone cooperation in acid resistance. *Nat. Chem. Biol.* **7**, 671-677 (2011).
7. Krojer, T., Garrido-Franco, M., Huber, R., Ehrmann, M. & Clausen, T. Crystal structure of DegP (HtrA) reveals a new protease-chaperone machine. *Nature* **416**, 455-459 (2002).
8. Krojer, T., Sawa, J., Huber, R. & Clausen, T. HtrA proteases have a conserved activation mechanism that can be triggered by distinct molecular cues. *Nat. Struct. Mol. Biol.* **17**, 844-852 (2010).
9. Ihara, K. et al. Crystal Structure of Human RhoA in a Dominantly Active Form Complexed with a GTP Analogue. *J. Biol. Chem.* **273**, 9656-9666 (1998).
10. Wong, K.-W., Mohammadi, S. & Isberg, R.R. Disruption of RhoGDI and RhoA Regulation by a Rac1 Specificity Switch Mutant. *J. Biol. Chem.* **281**, 40379-40388 (2006).
11. Lin, S. et al. Genetically Encoded Cleavable Protein Photo-Cross-Linker. *J. Am. Chem. Soc.* **136**, 11860-11863 (2014).
